# A 3’UTR-derived small RNA modulates the life cycle of the cholera toxin–encoding filamentous phage, CTXϕ

**DOI:** 10.1073/pnas.2535142123

**Published:** 2026-06-02

**Authors:** Anne Lippegaus, James R.J. Haycocks, Eoghan O’Driscoll, Marcel Sprenger, Kerstin Thriene, Elke-Martina Jung, Malte Siemers, Sebastian Krautwurst, David C. Grainger, Kai Papenfort

**Affiliations:** ^a^ Institute of Microbiology, General Microbiology, Friedrich Schiller University, Jena 07743, Germany; ^b^ Institute of Microbiology and Infection, School of Biosciences, University of Birmingham, Birmingham B15 2TT, United Kingdom; ^c^ Microverse Cluster, Friedrich Schiller University, Jena 07743, Germany

**Keywords:** small RNA, Hfq, RIL-seq, *Vibrio cholerae*, CTXϕ phage

## Abstract

Bacteriophages (phages) are well known to be one of the major driving forces in bacterial evolution. This also applies to virulent microorganisms, such as the major human pathogen *Vibrio cholerae*, whose pathogenic potential and epidemic proliferation largely depends on the interaction with environmental phages. Specifically, integration of the CTXϕ phage genome into the first chromosome of *V. cholerae* also introduced the *ctxAB* genes, encoding the primary toxin responsible for the severe acute diarrheal disease, cholera. Whereas the mechanisms underlying CTXϕ-associated horizontal gene transfer and transcriptional control of the *ctxAB* genes have been intensively studied over the past years, posttranscriptional regulation affecting the CTXϕ life cycle has not been documented. Here, we report the identification and characterization of the CisR small RNA (sRNA) that is produced from the 3’UTR (untranslated region) of the *prtV* gene and inhibits the expression of the CTXϕ-encoded *cep* mRNA. CisR-mediated repression of *cep* involves Hfq-assisted base-pairing of the two transcripts and results in reduced CTXϕ production under stress conditions. We further demonstrate that transcription of *prtV-cisR* requires both the master quorum-sensing regulator HapR and CRP (cAMP receptor protein), a global regulator of carbon metabolism. Taken together, our work provides evidence that *V. cholerae* employs sRNA-mediated posttranscriptional gene regulation to coordinate CTXϕ activation with both cell density and nutrient availability.

## Significance

The integration of the CTXϕ phage genome, which carries the *ctxAB* toxin genes, is essential for cholera pathogenesis in humans. While transcriptional control of CTXϕ and *ctxAB* has been well-studied, posttranscriptional mechanisms remain unexplored. Here, we identify and characterize CisR, a small RNA derived from the 3′ untranslated region of *prtV*, which inhibits the CTXϕ-encoded *cep* mRNA through Hfq-dependent base-pairing. CisR-mediated regulation limits phage production under stress conditions and is coregulated by the quorum-sensing factor HapR and the metabolic regulator CRP. Our findings reveal an RNA-based mechanism linking CTXϕ phage activation to cell density and nutrient status of *Vibrio cholerae*.

Cholera remains a significant health challenge in numerous regions of the developing world, accounting for an estimated ~2.8 million cases and ~90,000 fatalities annually (1). The causative agent, *Vibrio cholerae*, is ubiquitously present in marine environments, however, many *V. cholerae* strains are nonpathogenic or only cause sporadic instances of gastroenteritis (2). Pathogenicity of *V. cholerae* hinges on the acquisition of various virulence factors, particularly cholera toxin (CT) and the toxin-coregulated pilus (TCP). CT induces profuse watery diarrhea, a major contributor to cholera-related mortality and its rapid spread, while TCP facilitates colonization of the small intestine (3). The genes encoding CT, *ctxAB*, are carried in the genome of a lysogenic filamentous phage CTXϕ and genomic analyses of epidemic *V. cholerae* strains suggest multiple independent events of toxigenic conversion in the history of cholera (2, 4, 5).

Since its discovery (6), the CTXϕ life cycle has been investigated in great detail. After entering *V. cholerae* cells, the single-stranded genome of CTXϕ can either integrate into the chromosome of *V. cholerae* or convert into double-stranded DNA to facilitate rolling-circle replication and transcription of genes crucial for phage replication and morphogenesis (7). The lysogenic phase of CTXϕ is mainly regulated by the phage repressor protein, RstR, which inhibits the key phage mobilization genes, *rstA* and *rstB*. Activity of RstR is supported by the host-encoded LexA protein (the master regulator of SOS response) and thereby links CTXϕ activation to DNA damage (8). In addition, RstR activity is counteracted by RstC, an antirepressor protein located on the RS1 satellite phage (9). The core components of CTXϕ consist of seven genes: *ctxAB* (encoding CT subunits A and B) and five structural genes (*cep*, *psh*, *g^IIICTX^*, *ace*, and *zot*), which play crucial roles in phage morphogenesis and assembly (10). Similar to many other filamentous phages, CTXϕ secretion does not involve cell lysis, but rather depends on the type II secretion system (T2SS) of the host cell (11).

Besides LexA, several other host-encoded proteins have been reported to interact with CTXϕ at the genomic or regulatory level (12, 13). In this context, regulation of *ctxAB* regulation has been a major focus, reflecting the pivotal role of CT in cholera pathogenesis (14). Expression control of the *ctxAB* operon involves the ToxT transcriptional regulator that is encoded on another horizontally acquired genomic element, called VPI-1 (*Vibrio* pathogenicity island 1). ToxT activity is modulated by bile and fatty acids (15, 16) and, besides *ctxAB*, controls the transcription of additional virulence-related genes, including the TarA and TarB sRNAs. Whereas TarA has been reported to regulate glucose uptake (17), TarB inhibits the expression of the *tcpF* virulence factor and has been documented to participate in phage defense functions (18–20).

Both, TarA and TarB, belong to the large group of Hfq-binding sRNAs that have been studied in various pathogenic bacteria, including *V. cholerae* (21). Hfq functions as an RNA chaperone that promotes sRNA stability and facilitates base-pairing of sRNAs with target mRNAs (22, 23). A single sRNA usually controls multiple target genes and in some cases sRNAs have been documented to rival transcription factors with respect to the number of regulated targets (24). Thereby, sRNAs can promote stress responses and virulence, coordinate metabolic fluxes, and support overall cellular homeostasis (25–28). Traditionally, sRNA genes have been discovered in intergenic regions of the genome, however, global transcriptome analyses have now revealed that sRNAs can also correspond to coding sequences, as well as the 5’ and 3’UTRs of mRNAs (29).

In this manuscript, we identified a 3’UTR-derived sRNA from *V. cholerae* that is transcribed together with the *prtV* mRNA, encoding an extracellular protease involved in host–microbe interaction and biofilm formation (30, 31). Global RNA interactome studies revealed that the sRNA base-pairs with and inhibits the translation of multiple targets, including the *cep* mRNA of phage CTXϕ. Accordingly, we named the sRNA, CisR (CTXϕ inhibiting small RNA). Expression of the *prtV-cisR* transcript requires transcriptional activation by HapR and CRP, linking CisR accumulation to quorum sensing and general carbon metabolism, respectively. Mutation of *cisR* increased Cep protein levels under stress conditions and promoted CTXϕ production, suggesting modulation of CTXϕ phage levels. Taken together, our data indicate that the core-genome encoded CisR sRNA controls the production of the horizontally acquired virulence-associated gene that has a crucial role in the life cycle of the CTXϕ phage.

## Results

### Identification of Differentially Expressed sRNAs Under Virulence Inducing Conditions.

**Fig. 1.**
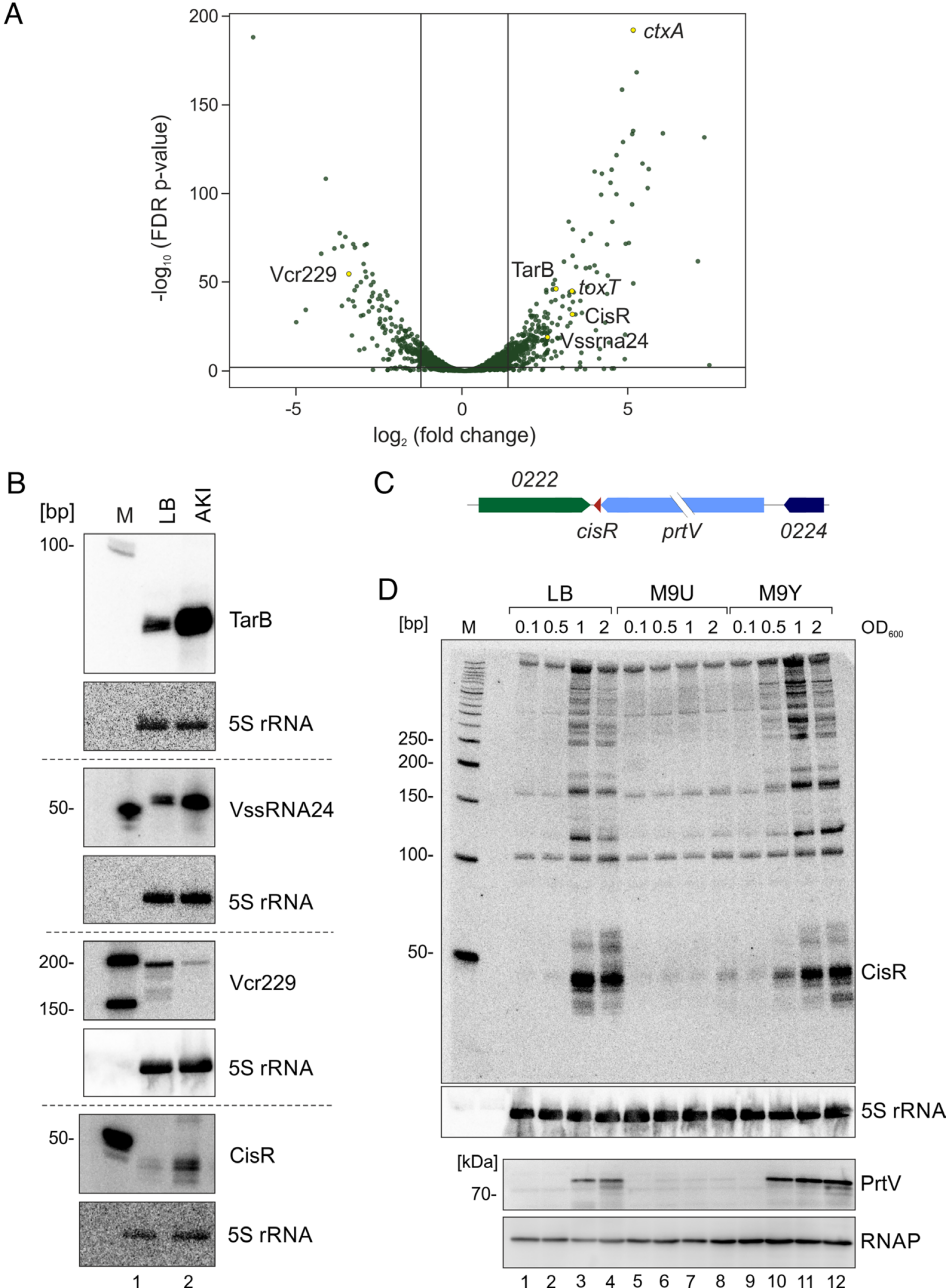
Transcriptomics under virulence-inducing conditions. (*A*) Volcano plot of genome-wide transcript changes in response to virulence inducing conditions (AKI medium vs. LB media). Lines indicate cut-offs of differentially regulated genes at threefold and FDR-adjusted *P*-value ≤ 0.01. Genes validated by qRT-PCR and northern blotting are marked in yellow. (*B*) Validation of differentially regulated sRNAs by northern blotting. RNA samples were obtained from *V. cholerae* WT cells cultivated in LB or AKI medium. sRNA levels were monitored by northern blotting and probed with specific oligonucleotides for the indicated sRNAs. 5S ribosomal RNA served as a loading control. (*C*) Schematic representation of the genomic context of *cisR*. *cisR* is marked in red and *prtV* in light blue. (*D*) Expression of CisR and PrtV. *V. cholerae* WT cells were cultivated in LB, M9U (glucose), and M9Y (glycerol) medium. RNA and protein samples were collected at various stages of growth. Northern blot analysis was performed to determine CisR levels and western blot analysis was performed using a PrtV-specific antibody. 5S ribosomal RNA and RNAP served as loading controls for western and northern blots, respectively.

Bacterial sRNAs are well known to be differentially expressed under various stress conditions, as well as when virulence gene expression is induced (32, 33). In *V. cholerae*, the TarA and TarB sRNAs have been reported to be activated by the ToxT transcriptional regulator, yet no systemic searches for virulence-associated sRNAs have been performed. To close this gap, we compared the gene expression profiles of wild-type (WT) *V. cholerae* cells cultivated in rich media (when virulence gene expression is repressed) with AKI growth conditions, a protocol known to activate virulence gene expression outside the human host (34). We confirmed efficient activation of virulence genes in AKI samples by quantifying the levels of the pathogenicity-associated *toxT* and *ctxA* mRNAs using qRT-PCR (quantitative real-time PCR). As expected, the expression of both transcripts was strongly upregulated under virulence-inducing conditions (*SI Appendix*, Fig. S1). We next performed high-throughput-sequencing to determine the global gene expression changes associated with growth under AKI growth conditions in *V. cholerae*. These analyses revealed dozens of differentially expressed sRNAs and mRNAs, including known virulence-associated transcripts, such as *toxT* and the TarB sRNA (Fig. 1*A*). Other sRNAs displaying differential expression involved VssRNA24 (35), which was upregulated under AKI conditions, as well as the uncharacterized Vcr229 sRNA (36) displaying reduced expression (Fig. 1 *A* and *B*). In addition, another uncharacterized sRNA, CisR [initially identified as Vcr096 (37)], was also upregulated under virulence-inducing conditions (Fig. 1 *A* and *B*). The *cisR* gene is located at the 3’ end of *prtV* (Fig. 1*C*), encoding a Zn^2+^-binding extracellular protease, which is required by *V. cholerae* to infect *Caenorhabditis elegans* (30).

To further investigate regulation of CisR in *V. cholerae*, we next probed its expression by northern blotting. Here, we detected several *cisR*-specific bands and that the sRNA accumulates as a ~50 nucleotide-long transcript (Fig. 1 *D*, *Top*). In complex media (LB), expression of CisR was increased at high cell densities, whereas cultivation of *V. cholerae* in minimal media containing glucose as the sole carbon source resulted in strongly reduced CisR levels. In contrast, minimal media supplemented with glycerol activated CisR expression at high cell densities, suggesting that *cisR* synthesis is affected by carbon utilization and cell density. We also probed PrtV protein levels under the same conditions using western blot analysis. These data showed that PrtV and CisR display highly similar expression patterns (Fig. 1 *D*, *Bottom*), suggesting that their expression might be coupled.

### CisR Is Produced from the 3’End of the *prtV* mRNA.

Previous work on *V. cholerae* sRNAs indicated that a large fraction of these regulators are processed from longer transcripts (36, 38). To address this question for *prtV*-*cisR*, we first aligned the *cisR* sequences from various *Vibrio* species, showing that the region downstream of the *prtV* stop codon as well as segments of the 3’ UTR (including a Rho-independent terminator element) are highly conserved (*SI Appendix*, Fig. S2*A*). In contrast, these analyses did not reveal any *cisR*-associated promoter element, supporting the hypothesis that the sRNA might be processed from the *prtV* mRNA. This idea was further supported by a previous study, indicating RNase E cleavage site at the 5’ end of *cisR* (39) (*SI Appendix*, Fig. S2*B*).

To further test if accumulation of CisR in *V. cholerae* results from RNase E-mediated cleavage of the *prtV-cisR* transcript, we tested CisR expression in the absence of active RNase E. Although RNase E is essential in *V. cholerae* and many other bacteria (21), its function can be studied in a temperature-sensitive mutant strain (*rne*TS), which allows for the selective inactivation of RNase E at nonpermissive temperatures (39). Specifically, we collected total RNA samples from WT and the *rne*TS strain at permissive (30 °C) and nonpermissive (44 °C) temperatures and compared CisR levels by northern blotting. Our results showed that the mature CisR sRNA was not detected in the absence of RNase E (*SI Appendix*, Fig. S2*C*), indicating that the endoribonuclease is indeed required for cleavage of the *prtV-cisR* transcript and CisR accumulation in the cell.

To further investigate the source of CisR expression in *V. cholerae*, we designed two plasmids: P_long_, which includes a predicted promoter region upstream of the adjacent gene *vca0224* (encoding a hypothetical protein) (38), and a truncated version, P_short_, starting from the *prtV* start codon (testing for potential internal promoters in the *prtV* coding sequence) (*SI Appendix*, Fig. S2*D*). We transferred both plasmids in *V. cholerae* Δ*cisR* cells and quantified CisR levels by northern blotting. Comparison of the CisR-specific signals with *V. cholerae* WT cells carrying an empty vector control showed increased CisR expression from plasmid P_long_, whereas the levels of CisR in P_short_ were down-regulated. Taken together, our data suggest that *cisR* is cotranscribed with *prtV* using a shared promoter element located upstream of *vca0224*.

### CisR Expression Is Activated by the HapR and CRP Transcription Factors.

**Fig. 2.**
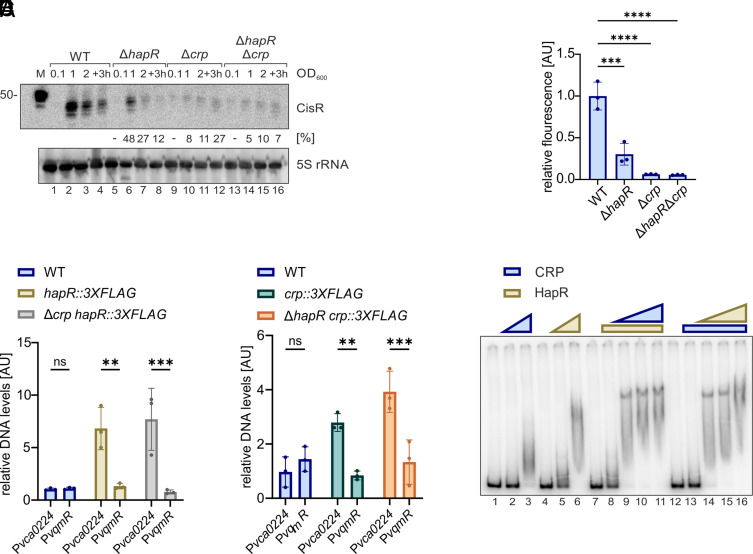
Transcriptional control of *cisR*. (*A*) Role of HapR and CRP for CisR levels. *V. cholerae* WT, Δ*hapR*, Δ*crp,* and Δ*hapR* Δ*crp* were cultivated in LB medium and RNA samples were collected at different stages of growth. Northern blot analysis was performed to determine CisR levels. Probing for 5S ribosomal RNA served as loading control. (*B*) Regulation of the promoter located in front of *vca0224* (P*vca0224*). *V. cholerae* WT, Δ*hapR*, Δ*crp,* and Δ*hapR* Δ*crp* carrying a GFP-based transcriptional reporter for *vca0224* (P*vca0224::GFP*) were cultivated in LB medium to OD_600_ of 1.0 and analyzed for fluorescence. *V. cholerae* WT were set to 1. Bars show mean of independent biological replicates ±SD, n = 3. For statistical analyses, an ordinary one-way ANOVA with Dunnett’s multiple comparison test was used (∗∗∗*P* ≤ 0.001, ∗∗∗∗*P* ≤ 0.0001). (*C*) ChIP analysis of HapR. *V. cholerae* WT, *hapR::3XFLAG* cells and *Δcrp hapR::3XFLAG* were cultivated to OD_600_ of 1.0 and subjected to chromatin immunoprecipitation (ChIP). Bar graphs show relative levels of *cisR* and *vqmR* promoters (P*vca0224* and P*vqmR*), determined by quantitative PCR and P*vca0224* levels in WT *V. cholerae* cells were set to 1. Data are presented as mean values of independent biological replicates ±SD, n = 3. For statistical analyses, a two-way ANOVA with the Šídák method for multiple comparisons was used (ns, not significant, ∗∗*P* ≤ 0.01, ∗∗∗*P* ≤ 0.001). (*D*) ChIP analysis of CRP. *V. cholerae* WT*, crp::3XFLAG* cells and Δ*hapR crp::3XFLAG* were cultivated to OD_600_ of 1.0 and subjected to chromatin immunoprecipitation (ChIP). Bar graphs show relative levels of the *cisR* and the *vqmR* promoters (P*vca0224* and P*vqmR*), determined by quantitative PCR and P*cisR* levels in WT *V. cholerae* were set to 1. Data are presented as mean values of independent biological replicates ±SD, n = 3. For statistical analysis, a two-way ANOVA with the Šídák method for multiple comparison was used (ns, not significant, ∗∗*P* ≤ 0.01, ∗∗∗*P* ≤ 0.001). (*E*) Electrophoretic mobility shift assay of HapR and CRP binding to P*vca0224*. Radiolabeled P*vca0224* transcript was incubated alone (lanes 1, 4, 7, and 12), with increasing concentrations (0.5 µM to 2 µM) of purified HapR (lanes 2 to 3) and CRP (lanes 5 to 6) or with 0.5 µM HapR or CRP while increasing the other one from 0.5 µM to 2 µM (lanes 8 to 11 and 13 to 16). Complexes were separated on a native polyacrylamide gel and visualized by autoradiography.

To identify factors controlling *vca0224*-*prtV*-*cisR* expression in *V. cholerae*, we searched for regulatory DNA sequences upstream of *vca0224*. Alignment of equivalent sequences from various *Vibrio* strains revealed conserved −35 and −10 promoter elements, as well as putative binding sites for HapR and CRP (40, 41) (*SI Appendix*, Fig. S3*A*). To investigate the roles of the two transcription factors on CisR expression, we constructed *V. cholerae* single-gene deletion mutants of *hapR* and *crp*, as well as a double mutant lacking both genes and measured CisR levels at various growth conditions using northern blot analysis (Fig. 2*A*). Compared to WT cells, the *hapR* mutant produced less CisR at all stages of growth, with residual levels in stationary phase (OD_600_ of 1.0). CisR levels were even further reduced in the *crp* mutant and when both transcriptional regulators were absent. We observed a similar regulatory pattern using a GFP-based transcriptional reporter of the promoter upstream of *vca0224* (Fig. 2*B*) and using qRT-PCR we confirmed that expression of the *prtV* mRNA is also regulated by HapR and CRP (*SI Appendix*, Fig. S3*B*). In summary, these data indicated that both HapR and CRP positively regulate *prtV*-*cisR* expression by controlling the promoter upstream of *vca0224*.

To further investigate the roles of HapR and CRP in CisR expression, we generated *hapR* and *crp* complementation plasmids (p-*hapR* and p-*crp*, respectively) and tested their effect on CisR levels in *V. cholerae* WT cells and the relevant mutant strains by northern blotting. Whereas both plasmids did not significantly change CisR expression in WT cells, p-*hapR* restored CisR expression in Δ*hapR* cells, but not in the Δ*crp* or Δ*crp*/hapR mutants (*SI Appendix*, Fig. S3*C*). Similarly, p-*crp* restored CisR levels in the Δ*crp* mutant. Interestingly, Crp was also able to up-regulate CisR expression in the absence of *hapR*, suggesting that CRP has key regulatory function for *cisR* (and *prtV*) synthesis.

We next sought to understand if the regulatory impact of HapR and CRP was direct. To this end, we used chromatin immunoprecipitation (ChIP) assays. Specifically, we generated *V. cholerae* strains producing chromosomally tagged HapR::3XFLAG or CRP::3XFLAG proteins and performed immunoprecipitation using anti-FLAG antibodies. The copurified DNA was analyzed by quantitative PCR. When compared to a nontagged WT control, coimmunoprecipitation of HapR::3XFLAG enriched the *vca0224* promoter sequence ~sevenfold, while CRP::3XFLAG enriched the same region ~threefold (Fig. 2 *C* and *D*), confirming that both HapR and CRP directly bind to the *vca0224* promoter in vivo. As a negative control, we tested coimmunoprecipitation of the unrelated *vqmR* promoter sequence, which showed no significant enrichment in either ChIP experiment. Importantly, binding of HapR to the *vca0224* promoter was observed in the absence of CRP, and likewise CRP binding occurred in the absence of HapR.

While ChIP analysis by PCR can detect binding of CRP and HapR, the method lacks the resolution to exactly map interactions and is only semiquantitative. Hence, using radiolabeled *vca0224* promoter DNA, and purified CRP and HapR, we did EMSA (electrophoretic mobility shift assay) experiments. In accordance with our ChIP data, both proteins were able to bind the promoter DNA individually. However, the bands corresponding to each protein–DNA complex were diffuse (Fig. 2*E*, lanes 3 and 6). This indicates weak or unstable binding. Conversely, when CRP and HapR were applied in combination, a focused low mobility band was apparent, even with minimal concentrations of each protein (Fig. 2*E*, lanes 9 and 14). Hence, binding of the factors is cooperative. Taken together, these results explain why full *prtV-cisR* expression requires both HapR and CRP (Fig. 2*A* and *SI Appendix*, Fig. S3*C*). Interestingly, we previously described an interaction, between CRP and HapR, required for cooperative binding at the *V. cholerae murQP* promoter (40). This interaction is lost with the CRP E55A substitution (CRP_E55A_). Further EMSAs showed that WT CRP, and CRP_E55A_, bound the *vca0224* promoter region and did not alter cooperative interactions with HapR (*SI Appendix*, Fig. S3*D*). This is not unexpected, since the arrangement of CRP and HapR binding sites is different at the *murQP* and *vca0224* promoters.

To better understand the interplay between CRP and HapR, we used in vitro DNase I footprinting (*SI Appendix*, Fig. S3*E*). In the absence of HapR, little change in the DNase I digestion pattern was induced by CRP (*SI Appendix*, Fig. S3*E*, lanes 1 to 3). This is consistent with our EMSA experiment that identified only unstable CRP binding (Fig. 2*E*). In the absence of CRP, HapR was able to footprint two distinct regions, but only at the highest protein concentration tested (*SI Appendix*, Fig. S3, lanes 4 to 6). We have named these sites HapR I (−173 to −149 bp) and HapR II (−131 to −105 bp). In both cases, the site is evidenced by a region of DNA protection (shown by colored bars in *SI Appendix*, Fig. S3*E*) and DNase I hypersensitivity at the upstream edge of the binding site (colored triangles). Consistent with our EMSA experiments, both CRP and HapR bound tightly in the presence of the other factor (*SI Appendix*, Fig. S3*E*, lanes 8 to 16). Thus, with low concentrations of HapR, CRP binds two regions. This is evidenced by both DNA protection and, as is typical for CRP, a series of DNase I hypersensitivity signals (shown by triangles in the figure) (42). These are caused by the sharp DNA bend induced by interaction with CRP (40). The two CRP binding regions are denoted CRP I (−200 to −178 bp) and CRP II (−149 to −131 bp) in *SI Appendix*, Fig. S3*E*. Consistent with cobinding, the DNA protection and DNase I hypersensitivity signals due to HapR are retained when CRP is present, with relative signal intensity depending on concentrations of each protein (*SI Appendix*, Fig. S3*E*, lanes 8 to 16). While CRP and HapR are bound unusually far upstream of the *vca0224* transcription start site (TSS) activation of transcription from such locations is not unprecedented (43). Hence, we used in vitro transcription assays, with purified *V. cholerae* RNA polymerase, and the regulatory factors (*SI Appendix*, Fig. S3*F*) to test for activation. Consistent with our in vivo and in vitro data (*SI Appendix*, Figs. S3 *B*–*E*), CRP alone activated transcription from the *vca0224* promoter, whereas HapR alone had no effect. However, maximal transcription only occurred in the presence of both proteins. Thus, CRP and HapR must activate *vca0224* directly. This explains why transcription of *cisR* (and *prtV*) requires both CRP, HapR, and associated metabolic and cell density signals.

### RIL-Seq Analysis Reveals the Hfq-Mediated RNA Interactome Under Virulence Conditions.

**Fig. 3.**
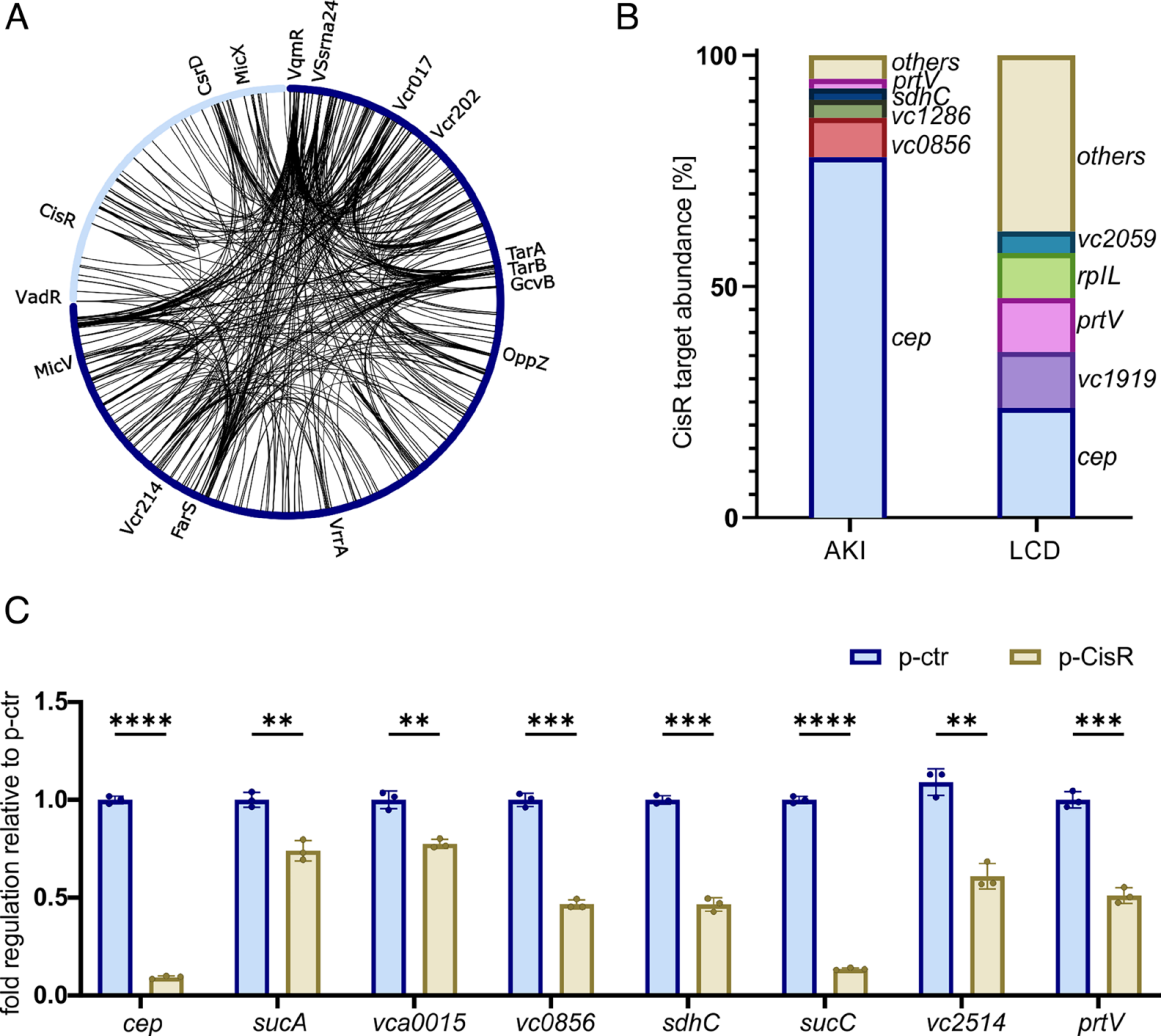
RIL-seq in *V. cholerae* under virulence inducing conditions. (*A*) Top 500 chimeras detected by RIL-seq under virulence inducing conditions. The first and the second chromosomes are marked in dark and light blue, respectively. The circos plot was generated using the circos component of the Dash Bio package. (*B*) Comparison of CisR target abundance in two Hfq::3XFLAG RIL-seq datasets, virulence conditions (AKI) and low cell density (LCD) (48). The total amount of recorded CisR—mRNA interactions were set to 100% for each condition, respectively (LCD: 215 and AKI: 331). The top 5 interaction partners are named individually; remaining interaction partners are summarized as others. (*C*) Validation of CisR targets predicted by RIL-seq. Translational GFP reporter fusions were cotransformed with a constitutive CisR expression plasmid (golden bars) or an empty control plasmid (blue bars) in *Escherichia coli* Top 10 cells. GFP production was measured and fluorophore levels from the control strains were set to 1. Error bars represent the SD of three independent biological replicates. Statistical significance was calculated using an unpaired *t* test (∗∗*P* ≤ 0.01, ∗∗∗*P* ≤ 0.001, ∗∗∗∗*P* ≤ 0.0001).

We next aimed to study the regulatory role of CisR in *V. cholerae.* To this end, we employed RIL-seq (RNA-interaction-by-ligation-and-sequencing) (44, 45) to determine the global Hfq-mediated RNA–RNA interactome under virulence conditions. Using *V. cholerae* cells expressing Hfq::3XFLAG protein from its native chromosomal locus, we cultivated cells under AKI conditions (36). RIL-seq involves UV crosslinking and the ligation of RNA–RNA pairs bound by Hfq, followed by high-throughput sequencing of the resulting chimeric reads. This method thus provides a global map of Hfq-dependent interactions. A *V. cholerae* WT strain lacking the 3XFLAG epitope served as a negative control to ensure specificity of these interactions. Our analysis captured 1,395 RNA–RNA chimeras interacting with Hfq (Fig. 3*A*). Under virulence-inducing conditions, RIL-seq revealed that the vast majority (~80%) of the CisR interactions involved RNA–RNA duplex with *cep* (core encoded pilus), which encodes a core structural element of the CTXϕ phage (46, 47). In contrast, under standard laboratory conditions (48), this interaction accounted only for ~20% of CisR interactions (Fig. 3*B*). These findings highlight the dynamic nature of RNA networks in response to different growth conditions and suggested that CisR might regulate CTXϕ-encoded genes.

To confirm the regulatory role of CisR, we employed a GFP-based reporter system (49, 50) to monitor posttranscriptional control of eight potential targets (selected based on their abundance in RIL-seq experiments). In this system, one plasmid contains the 5’ untranslated region (5’ UTR) and the sequence encoding the first 20 amino acids of the target genes fused to *gfp*, whereas the sRNA is expressed from a second plasmid. In both cases, transcription is driven from constitutive promoters, thus specifically monitoring posttranscriptional gene regulation. We validated stable CisR expression in *E. coli* (*SI Appendix*, Fig. S4*A*) and using the ChimericFragments workflow (51) and the IntaRNA algorithm (52), we were able to predict RNA duplex formation of CisR with the putative targets (*SI Appendix*, Figs. S4 *B*–*H*). Indeed, this strategy allowed us to validate regulation of all eight targets by CisR, of which *cep* showed the strongest repression (Fig. 3*C*). Additional targets included *sucA* (2-oxoglutarate decarboxylase)*, vca0015* (hypothetical protein)*, vc0856* (DnaJ chaperone)*, sdhC* (succinate dehydrogenase subunit)*, sucC* (succinyl-CoA synthetase subunit)*, vc2514* (UDP-N-acetylglucosamine enolpyruvyl transferase), and also *prtV*, indicating that CisR could inhibit the expression of its own operon. To test this, we introduced point mutations in CisR and *prtV::gfp* to probe the predicted RNA duplex (*SI Appendix*, Fig. S4 *B* and *I*). Mutation of CisR abrogated regulation of *prtV::gfp* and, *vice versa*, mutation of the *prtV* 5’UTR blocked regulation by WT CisR. In contrast, regulation was restored when both mutated variants were combined, indicating that CisR base-pairs with the 5’UTR of *prtV* and supporting the hypothesis that it acts as a feedback regulator. Of note, a similar regulatory mechanism has previously been observed for the 3’UTR-derived OppZ and CarZ sRNAs in *V. cholerae* (39).

### CisR Base-Pairs with the *Cep* mRNA Encoded by CTXΦ.

**Fig. 4.**
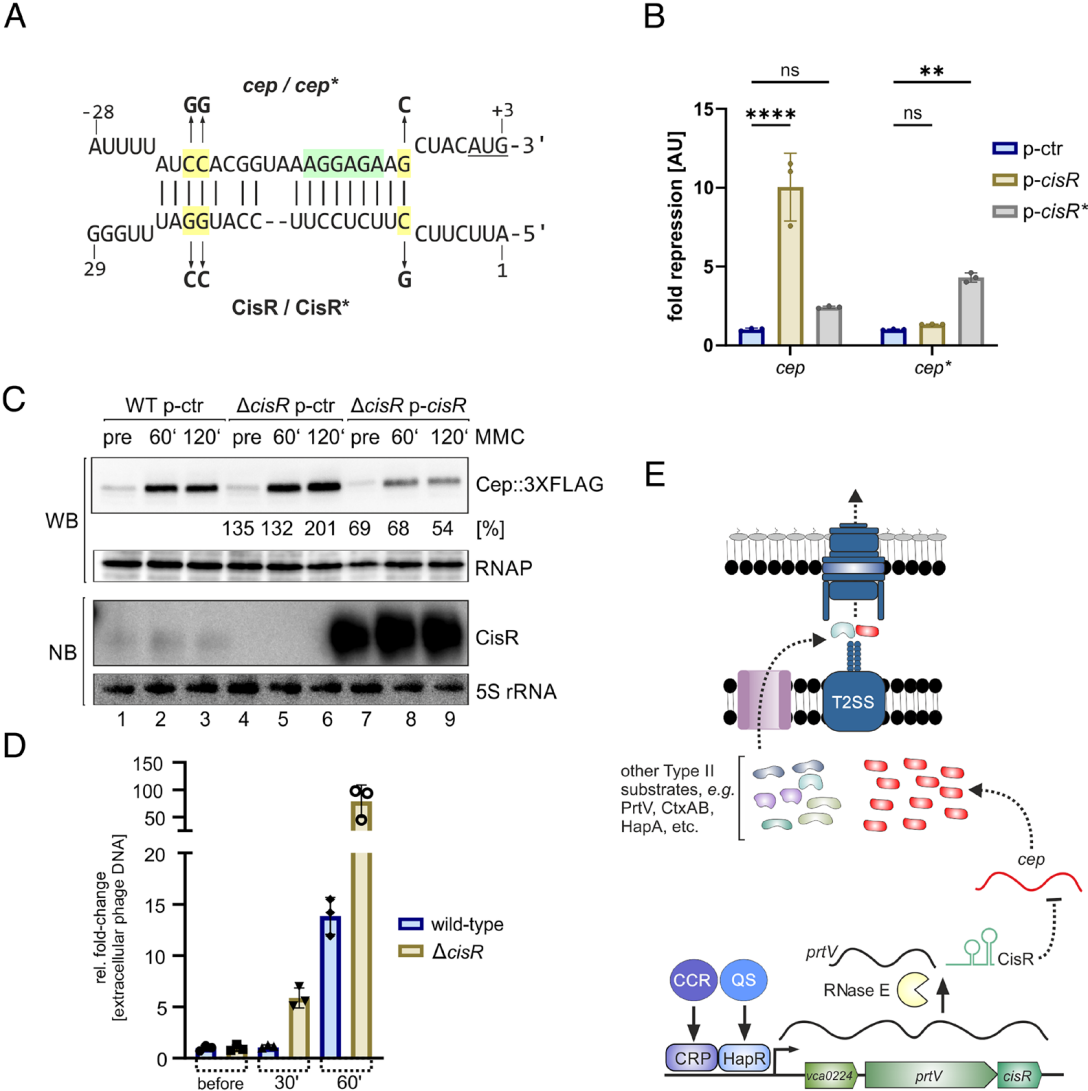
CisR inhibits Cep expression and CTX phage production. (*A*) Predicted base-pairing regions between CisR and *cep* by ChimericFragments (51). SD-sequence, start codon, and point mutations used in (*B*) are highlighted. (*B*) Compensatory mutation CisR and *cep*. Translational GFP reporter fusion of *cep*/cep* were cotransformed with constitutive CisR/CisR* expression plasmids or an empty control plasmid in *E. coli* Top 10 cells. GFP production was measured, fluorophore levels from the control strains were set to 1 and the fold repression was plotted. For statistical analyses, a two-way ANOVA with Dunnett’s multiple comparison test was used (ns, not significant, ∗∗*P* ≤ 0.01, ∗∗∗∗*P* ≤ 0.0001). (*C*) Effect of MMC on CisR and Cep. Protein and RNA samples of *V. cholerae cep::3XFLAG* p-ctr, Δ*cisR cep::3XFLAG* p-ctr and Δ*cisR cep::3XFLAG* p-*cisR* were collected in AKI medium pre, 60 min and 120 min post treatment with MMC (250 ng/mL). Protein levels of Cep::3XFLAG were monitored by western blotting and CisR RNA levels were monitored by northern blotting. RNAP and 5S ribosomal RNA served as loading controls for western and northern blot, respectively. (*D*) RstC-mediated activation of CTXϕ. Extracellular CTXϕ DNA levels were measured in cell-free supernatants of *V. cholerae* WT and Δ*cisR* cells carrying a pBAD-RstC plasmid using qPCR. DNA levels were quantified before and 30 and 60 min after RstC activation and normalized to the chromosomal *hfq* gene to control for potential cell lysis. The levels of CTXϕ DNA before pBAD induction were set to 1 and the relative production of extracellular CTXϕ phage DNA in WT and Δ*cisR* cells was plotted. (*E*) Regulatory model for CisR-mediated regulation of *cep*. Expression of the *vca0224-prtV-cisR* transcript is activated by the HapR and CRP transcription factors by quorum sensing (QS) and carbon catabolite repression (CCR). The transcript is processed by RNase E to release the CisR sRNA, which base-pairs with the *cep* mRNA to inhibit its translation. We speculate that high Cep levels might interfere with the type II-mediated secretion of other proteins in *V. cholerae* (e.g., PrtV, CtxAB, and HapA).

The above results suggested that the *cep* repression is the main role of CisR under virulence-inducing conditions (Fig. 3 *B* and *C*). To pinpoint the base-pairing position between CisR and *cep*, we probed the predicted RNA duplex by introducing point mutations at three critical positions (Fig. 4*A*). Mutation of CisR decreased its expression by ~2.5-fold (*SI Appendix*, Fig. S5*A*) and strongly impaired the sRNA’s ability to inhibit the *cep::gfp* reporter (Fig. 4*B*). Likewise, mutation of *cep* abrogated repression by CisR, however, combination of both mutated CisR and *cep* variants mostly restored regulation. These results confirmed the predicted RNA duplex and given that CisR interacts with the *cep* ribosome binding site, we predict that base-pairing results in inhibition of translation initiation at the *cep* mRNA.

The Cep protein constitutes the main major coat protein of CTXϕ and is required for virion morphogenesis (6, 53). We therefore asked if CisR-mediated repression of *cep* also affects the production of CTXΦ when phage replication is induced. To this end, we cultivated *V. cholerae* under AKI conditions and used mitomycin C (MMC) to prompt LexA-mediated CTXΦ activation (8, 54). Using qRT-PCR, we quantified *cep* mRNA levels in WT, Δ*cisR*, and CisR overexpression strains. In the absence of MMC, we observed only basal *cep* expression and *cisR* mutation or overexpression only modestly affected *cep* levels (*SI Appendix*, Fig. S5*B*). In contrast, MMC treatment strongly activated *cep* expression (>60-fold) and this effect was further enhanced in cells lacking *cisR* (>100-fold). In accordance with our hypothesis, overexpression of CisR reduced *cep* expression when compared to WT and Δ*cisR* cells. To corroborate these findings at the protein levels, we added a 3XFLAG epitope tag to the chromosomal *cep* gene and monitored Cep::3XFLAG production in the presence and absence of MMC and/or CisR. Analogous to *cep* mRNA levels, Cep::3XFLAG levels were drastically increased by MMC treatment and this effect was more pronounced in the Δ*cisR* mutant, when compared to isogenic WT cells (Fig. 4*C* and *SI Appendix*, Fig. S5*C*). As expected, overexpression of CisR inhibited Cep::3XFLAG expression in the absence and presence of MMC. Taken together, our data indicate that CisR plays a regulatory role in modulating Cep levels during phage induction and thus might affect the CTXϕ life cycle.

To test the latter, we measured CTXϕ phage DNA in *V. cholerae* cell supernatants by quantitative PCR (qPCR) in the presence and absence of *cisR* using two complementary experimental designs. Analogous to the previous setup, we first monitored extracellular CTXϕ phage DNA levels following MMC induction in WT, Δ*cisR*, and CisR overexpressing cells. In accordance with the RNA and protein data (*SI Appendix*, Figs. S5*A* and S4*C*), overexpression of CisR resulted in reduced CTXϕ phage DNA levels, whereas mutation of *cisR* had the inverse effect when MMC was added (*SI Appendix*, Fig. S5*D*). For the second approach, we harnessed the activity of the antirepressor RstC, which inhibits the phage repressor RstR and activates the transcription of genes required for CTXϕ production (9). Of note, DNA damage-induced CTXϕ activation has been reported to be highly heterogeneous in *V. cholerae* populations secreting only one phage particle per ~10 to 100 host cells (55). In contrast, induction of the *rstC* gene from the inducible pBAD promoter resulted in strongly elevated extracellular CTXϕ phage DNA levels (when compared to MMC induction, *SI Appendix*, Fig. S5*D*) and this effect was further pronounced in Δ*cisR* cells when compared to the isogenic WT strain (Fig. 4*D*). Taken together, these data suggest that CisR limits CTXϕ production by inhibiting Cep synthesis.

## Discussion

Filamentous phages play a crucial role in the evolution and pathogenicity of *Vibrio* species, particularly *Vibrio cholerae* (53, 56). The CTXϕ phage, which carries the cholera toxin (*ctxAB*) and zonula occludens toxin (*zot*), transforms nontoxigenic into toxigenic strains, facilitating pathogen dissemination through diarrhea (13, 57). Given the importance of the CTXϕ phage for the evolution and life cycle of *V. cholerae*, the mechanisms underlying phage genome integration and replication have been studied in great detail over the past few years (10, 47). In contrast, less is known about the interplay of the CTXϕ prophage with its *V. cholerae* host, focusing mainly on transcriptional regulation of the *ctxAB* toxin genes (58). In this study, we provide evidence that posttranscriptional regulation is also relevant for CTXϕ biology and its dissemination under stress conditions.

The Cep protein is a homolog of protein pVIII gene of filamentous coliphage Ff and related phages and constitutes the major coat protein of the CTXϕ (59, 60). Cep and pVIII play a crucial role in forming the viral capsid and it has been estimated that phage Ff requires ~2,700 copies of pVIII protein per virion (53). Thus, reducing Cep levels in the cell, e.g., by CisR-mediated repression, might provide an efficient mechanism to inhibit CTXϕ genome packaging and limit phage propagation. This process could help to coordinate CTXϕ replication with the nutrient status of the cell and reduce the negative impact of CTXϕ activation under stress conditions.

Unlike tailed phages, the larger size of filamentous phages prevents their assembly inside the cell (59). Instead, the virion assembles at the bacterial cell envelope, where the maturing phage is actively released through the cell envelope without lysing the host cell. Specifically, CTXϕ employs the EspD secretin of the Type II secretion system (T2SS) for phage release (11). It is therefore possible that CTXϕ activation and the associated surge of Cep proteins in the cell blocks the T2SS-mediated export of other proteins causing membrane stress and reduced fitness (Fig. 4*E*). Of note, CTXϕ also uses T2SS to release CT into the host environment and the PrtV protease, which is coproduced with CisR (Fig. 1 *C* and *D*), is exported through this pathway as well (61, 62). CisR also inhibits *prtV* expression (Fig. 3*C*), suggesting an additional level of feed-back regulation that could balance PrtV overproduction in the context of stress or as a result of transcriptional bursting (39).

Transcription of *vca0224,* and the downstream *prtV-cisR* genes, requires both HapR and CRP (Fig. 1*C* and *SI Appendix*, Fig. S2*D*). It is currently not known if *vca0224* constitutes a translated open reading frame, however, homologous proteins have been annotated in various other *Vibrio* species (63). The relevant promoter upstream of *vca0224* contains binding sites for CRP and HapR (*SI Appendix*, Fig. S2*A*) and binding of one of the transcription factors facilitates the recruitment of the other (Fig. 2*E* and *SI Appendix*, Fig. S3*D*). Coregulation of transcription by CRP and HapR has previously been observed in *V. cholerae* and has been studied at the molecular level for the *murPQ* promoter (40). In contrast to the *vca0224* promoter (*SI Appendix*, Fig. S3*A*), HapR and CRP bind overlapping DNA sequences upstream of *murPQ*, which results in HapR-mediated inhibition of CRP-dependent transcription. In addition, binding of CRP and HapR at the *murPQ* promoter requires direct protein–protein interaction via the negatively charged E55 surface residue of CRP. Consistent with different juxtaposition of the DNA bound proteins, this surface is not needed for HapR and CRP cooperativity at the *vca0224* promoter (*SI Appendix*, Fig. S3*D*). While we do not exclude direct interaction of CRP and HapR, upstream of *vca0224*, we speculate that cooperativity might also be facilitated by changes in DNA bending. However, as the focus of this work was to understand the function of CisR, we have not tested this further. As noted above, CRP, and other activators, can stimulate transcription when bound distant with respect to the TSS. Complex regulator binding arrays, far upstream of the TSS, can also maneuver activators into low affinity targets adjacent to the core promoter (64, 65). In the latter regard, we note a weak CRP site at position −61.5 with respect to the *vca0224* TSS (*SI Appendix*, Fig. S3 *A* and *E*; labeled putative CRP site III). We detect only minor changes to the DNase I digestion pattern in this location. Even so, we note that CRP can activate transcription by binding cooperatively with, rather than independently of, RNAP (66). Hence, occupation of CRP sites in DNase I footprints, required for activation, is not always apparent (66). In summary, the data support our prior conclusion that joint transcriptional regulation by CRP and HapR is common in *V. cholerae* [and likely other *Vibrios*, (67)], however, depending on how the promoter elements are organized, the interaction between the two transcription factors can either activate or repress gene expression.

Joint regulation of *cisR* by CRP and HapR could also provide important insights into potential additional physiological roles of the sRNA. Whereas HapR levels in *V. cholerae* are tightly regulated by quorum sensing (68), activation of CRP occurs in the absence of the preferred carbon source (i.e., glucose) through carbon catabolite repression (CCR) (69). Indeed, expression of CisR is strongly repressed at low cell densities or in the presence of glucose (Fig. 1*D*). This expression pattern might also provide a regulatory link to other CisR targets (e.g.,*sucA*, *sdhC*, *sucC*, *vc2514*, and *vca0015*; Fig. 3*C*), as these have been previously associated with quorum sensing and/or carbon metabolism (70, 71). Interestingly, *cep* is the only horizontally acquired gene in the list of CisR targets, which raises interesting questions about the establishment and evolution of the associated base-pairing interactions (72). Given that *cisR* transcription is linked to *prtV* (*SI Appendix*, Fig. S2*B*) and that CisR also inhibits *prtV* (Fig. 3*C*), we speculate autoregulation of *prtV* might constitute a conserved CisR function, whereas regulation of *cep* was added to the regulon at a later evolutionary stage when the CTXϕ phage integrated into the *V. cholerae* genome. This hypothesis is supported by our conservation studies, showing that *cisR* is conserved in various *Vibrio* species (*SI Appendix*, Fig. S2*A*), however, these species typically do not encode the CTXϕ phage. Indeed, a CisR homolog from *Vibrio mimicus*, which does not encode the CTXϕ phage, efficiently repressed the *cep::gfp* reporter (*SI Appendix*, Fig. S5*E*), whereas CisR from the more distantly related *Vibrio anguillarum* (*SI Appendix*, Fig. S2*A*) failed to show this regulation. These data suggest that the CisR base-pairing sequence required to control *cep* evolved in various *Vibrio* strains but independently of CTXϕ phage integration into the genome of *V. cholerae*. Of note, other interactions between horizontally acquired genes and core genome-encoded sRNAs have been previously described (73). One example is the highly conserved SgrS sRNA from *Salmonella enterica*, which base-pairs with and inhibits the translation of the *sopD* virulence factor mRNA (74). Likewise, horizontally acquired sRNAs, e.g., encoded on phages and prophages, have been shown to regulate host mRNAs (75). For instance, the VpdS and Lpr1 sRNAs from vibriophage VP882 and coliphage λ, respectively, both base-pair with host-encoded targets and thereby facilitate phage replication (20, 76). Taken together, these data indicate that sRNA-mediated control of phage life cycles might be more prevalent than previously expected.

## Methods

### Bacterial Strains and Growth Conditions.

Bacterial strains used in this study are listed in *SI Appendix*, Table S1. *V. cholerae* and *E. coli* cells were grown in LB, AKI, M9U, and M9Y media, as specified. The WT strain *V. cholerae* C6706 was used throughout the study. Unless otherwise stated, standard laboratory conditions consisted of cultures grown in LB medium at 37 °C with an aeration at 200 rpm. For specific experiments, *V. cholerae* C6706 cells were grown in AKI medium with 1 h of aeration (34) to induce CT expression in vitro. Cultures were compared to cells grown under standard laboratory conditions and harvested at an OD_600_ of 0.7. Chromosomal mutations in *V. cholerae* were generated using the pKAS32 suicide plasmid for allelic exchange (77). Plasmids were introduced into *V. cholerae* by RK2/RP4-based conjugal transfer from *E. coli* S17λpir plasmid donor strains (78). Where appropriate, media were supplemented with antibiotics at the following concentrations: 100 µg/mL ampicillin; 20 µg/mL chloramphenicol; 50 µg/mL kanamycin; 50 U/mL polymyxin B; 5 mg/mL streptomycin.

### Plasmids and DNA Oligonucleotides.

All plasmids and DNA oligonucleotides used in this study are listed in *SI Appendix*, Tables S2 and S3, respectively.

### Plasmid Construction.

All plasmids and all DNA oligonucleotide sequences are listed in *SI Appendix*, Tables S2 and S3, respectively. GFP reporter fusions were constructed as previously described (79). Inserts were amplified from *V. cholerae* genomic DNA with the respective oligonucleotide combinations indicated in the following and cloned into linearized pXG-10 (KPO-1702/−1703) via Gibson assembly (GA) (80): pAL42 (KPO-08335/KPO-08336), pAL72 (KPO-09376/KPO-09377), pAL76 (KPO-09384/KPO-09385), pAL75 (KPO-09382/KPO-09383), pSM003 (KPO-05249/KPO-05250), pAL73 KPO-09378/−09379. For pNP83 (KPO-03362/KPO-03363), pXG10 and corresponding insert were digested with NsiI and NheI and ligated. Plasmids pAL84 and pMS384 were obtained by site-directed mutagenesis of pAL42 (KPO-10106/KPO-10107) and pNP83 (KPO-12169/KPO-12170), respectively. For the sRNA expression plasmid pAL41, the insert was amplified from *V. cholerae* genomic DNA (KPO-08333/−08334) and the pEVS backbone (81) was linearized with KPO-0092 and KPO-1397. Plasmids pAL85, pAL52, pMS390, and pMS391 were obtained by site-directed mutagenesis of pAL41 using KPO-10158/KPO-8767, KPO-8766/KPO-8767, KPO-12217/KPO-12218, KPO-12219/KPO-12220 respectively. For pKAS32 plasmids (77) pAL48, pAL55, pKV126, pMS382, and pMS383 backbone was linearized with KPO-0267 and KPO-0268, and inserts were fused using GA. Up and down flanks were amplified from *V. cholerae* genomic DNA with KPO-8386/KPO-8387/KPO-8388/KPO-8389 (pAL48), KPO-8418/KPO-8419/KPO-8420/KPO-8421 (pAL55), KPO-12115/KPO-12116/KPO-12117/KPO-12123 (pMS382), KPO-12159/KPO-12160/KPO-12161/KPO-12162 (pMS383). For pKV126 up flank, tag sequence and down flank were amplified with KPO-06040/KPO-06041/KPO-06042/KPO-06043/KPO-06044/KPO-06045. Up and down flank were amplified from *V. cholerae* gDNA and the tag sequence was amplified from *V. cholerae hfq*::3XFLAG (KPS-0995). For pAL79, pMD0080 (71) was linearized with pBAD-ATGrev and pZE-Stop-Xbal, the insert was amplified from *V. cholerae* genomic DNA (KPO-9649/KPO-9650) and fused using GA. To obtain pDD11, the pCMW-1 (82) was linearized using KPO-2757/KPO-02758 and the insert was amplified from *V. cholerae* gDNA with KPO-02759/KPO-02760 and cloned via GA. To generate pAL50 the insert was amplified from *V. cholerae* gDNA with KPO-8384/KPO-8385 and GA was used to insert the PCR product into linearized pMD004 (39) (KPO-0196/KPO-1397). pAL17 was linearized with KPO-1953/KPO-2757 and the inserts to obtain pAL59, pAL60 were amplified from *V. cholerae* gDNA: KPO-8924/KPO-8926 (pAL59), KPO-8925/−8926 (pAL60) and fused using GA. To obtain pAL82, pRH17 was linearized with KPO-1952/KPO-1953, the insert was amplified from *V. cholerae* gDNA with KPO-9800/ KPO-9801 and fused using GA. The P*vca*0224 fragment used for in vitro experiments was amplified using primers P*vca0224* F/P*vca0224* R. The resulting fragment was cloned *Eco*R1-*Hin*dIII into plasmid pSR (83).

#### RNA isolation, northern blot analysis, and qRT-PCR.

Total RNA was prepared and blotted as described previously (50). Nylon-membranes (Sigma #15356) were hybridized with (^32^P) labeled DNA oligonucleotides at 42 °C or with riboprobes at 63 °C. Riboprobes were generated using the MAXIscript T7 Transcription Kit (ThermoFisher, scientific, #AM1312). Signals were visualized using an Amersham Typhoon phosphoimager (GE Healthcare) and quantified with GelQuant software (BiochemLabSolutions). Oligonucleotides for northern blot analysis are provided in *SI Appendix*, Table S3. For qRT-PCR, total RNA was DNA-digested using TURBO^TM^ DNase (ThermoFisher scientific, #AM2238). qRT-PCR was performed using the Luna® Universal One-Step RT-qPCR Kit (New England Biolabs, #E3005). *hfq* was used as reference gene. Oligonucleotides used in all qRT-PCR analyses are also provided in *SI Appendix*, Table S3.

#### Fluorescence measurements.

Fluorescence assays to measure GFP expression were conducted as previously described (49). *E. coli* Top 10 cells were cultivated overnight in LB medium. To measure promoter activity, *V. cholerae* strains carrying P*vca0224*::GFP transcriptional reporter were cultivated in LB medium and samples were collected at indicated time-points. For all fluorescence measurements, three independent replicates were used for each strain. Cells were washed and resuspended in PBS and relative fluorescence was determined using a Spark 10 M plate reader (Tecan). Control samples not expressing fluorescent proteins were used to subtract background fluorescence.

#### Western blot analysis.

Experiments were performed as previously described (71). Denatured protein samples were separated using SDS-PAGE and transferred to PVDF membranes for western blot analysis. 3XFLAG-tagged fusions were detected using anti-FLAG antibody (Sigma, #F1804), PrtV was detected using a previously described antibody (30). RnaPα served as a loading control and was detected using anti-RnaPα antibody (BioLegend, #WP003). Signals were visualized using a Fusion FX EDGE imager (Vilber) and band intensities were quantified using BIO-1D software (Vilber).

#### RstC-mediated CTXϕ activation.

*V. cholerae* WT and Δ*cisR* cells carrying pBAD::RstC were grown to early stationary phase (OD_600_ of 1.0) and phage DNA was isolated from cell supernatants before and after the addition of L-arabinose (0.2% final conc.). qPCR was employed to quantify extracellular CTXϕ phage DNA (*cep* gene). *Cep* originating from the integrated phage on the chromosome may be present as a result of cell lysis. This was controlled for by measuring the levels of the chromosomally encoded *hfq* gene. The relevant oligonucleotides used for qPCR are listed in *SI Appendix*, Table S3.

#### MMC*-*mediated CTXϕ activation.

*V. cholerae* WT and Δ*cisR* cells carrying the pCMW-1 control plasmid as well as a strain carrying p*cisR* were grown to early stationary phase (OD_600_ of 1.0). Phage DNA was isolated from cell supernatants before and after the addition of 200 ng/mL of Mitomycin C (Sigma Aldrich, #M0503) at 1 and 2 h after the induction. This was done by spinning down 2 mL of culture at full speed 4 °C then filter sterilizing the supernatant using 0.22 µm PVDF syringe filter (Carl Roth, # XE04.1). The phage DNA isolation kit (Norgen Biotech, # 46800) was then used including the optional step of a DNase I digest (New England Biolabs, #M0303S) and Proteinase K (New England Biolabs, # P8107S) after the DNase I digest and inactivation 1 ng of pUC19 was spiked in to assist with standardizing the concentration before the qPCR was run. The GoTaq qPCR master mix was used for qPCR (Promega, #A6001). The relevant oligonucleotides used for qPCR are listed in *SI Appendix*, Table S3.

#### Transcriptomic analysis using RNA-seq.

The *V. cholerae* WT strain was cultivated in AKI and LB media, as described above. Total RNA was isolated and DNase digested (ThermoFisher scientific, #AM2238). RNA integrity was assessed using an Agilent 2100 Bioanalyzer. cDNA libraries were prepared with the NEBNext® Multiplex Small RNA Library Prep Set for Illumina (New England Biolabs, #E7300) and cDNA library quality was confirmed on an Agilent 2100 Bioanalyzer. Pooled cDNA libraries were sequenced on a NextSeq1000 system with a 100-nt read length in single-read mode. Demultiplexed raw reads were trimmed for quality and adaptor sequences, then mapped to the *V. cholerae* reference genome (NCBI accession numbers NC_002505.1, NC_002506.1) using CLC Genomics Workbench (Qiagen) with standard parameter settings. Fold enrichment under virulence inducing conditions was compared to standard laboratory conditions using the CLC “Differential Expression for RNA-Seq” tool. Genes with a fold change ≥3 and an FDR-adjusted *P*-value ≤0.01 were defined as differentially expressed.

#### ChIP and quantitative PCR.

*V. cholerae* WT, *hapR::3xFLAG*, Δ*crp hapR::3xFLAG*, *crp::3xFLAG*, and Δ*hapR crp::3xFLAG* strains were cultivated to OD_600_ of 1.0 in LB medium. ChIP experiments to determine transcription factor binding were performed as described previously (48). Briefly, cells were cross-linked with formaldehyde (1% final concentration) and lysed in FA lysis buffer (50 mM HEPES-KOH pH = 7, 150 mM NaCl, 1 mM EDTA, 1% Triton X-100, 0.1% Sodium deoxycholate, 0.1% SDS), supplemented with 4 mg/mL lysozyme. Cross-linked lysates were sonicated (2 × 30’ pulses) and subjected to immunoprecipitation using Protein A Sepharose beads (Sigma, #IP02) and anti-FLAG antibody (Sigma, #F3165) for 90 min. Following stringent washes with ChIP wash buffer (10 mM Tris-HCl pH = 8, 250 mM LiCl, 1 mM EDTA, 0.5% Nonidet-P40, 0.5% Sodium Deoxylate), DNA–protein complexes were eluted in ChIP elution buffer (50 mM Tris-HCl pH = 7.5, 10 mM EDTA, 1% SDS) and boiled for 10 min to reverse DNA–protein cross-links. DNA was purified by phenol-based extraction. Quantitative PCR (qPCR) was performed using GoTaq® qPCR Master Mix (Promega, #A6002) and the CFX96 Real-Time PCR System (Bio-Rad), with *recA* as the reference gene. The oligonucleotides used for qPCR are listed in *SI Appendix*, Table S3.

#### Purified proteins.

*V. cholerae* CRP, HapR, RNA polymerase, and σ^70^ proteins were purified as previously described (40).

#### EMSA and DNase I footprinting.

Promoter DNA fragments were excised from plasmid pSR, end-labeled with γ^32^-ATP, and treated with T4 PNK (New England Biolabs, #M0201). EMSAs and DNase I footprinting experiments were performed as described previously (84). For both experiments DNA and proteins were incubated in buffer containing 40 mM Tris acetate pH 7.9, 50 mM KCl, 5 mM MgCl2, 500 μM DTT, and 12.5 μg/mL Herring Sperm DNA at 37 oC for 15 min (concentrations of proteins used as detailed in figure legends). Where CRP was used, this was preincubated with 0.25 mM cAMP before addition to reactions. EMSA reactions were analyzed on a 7.5% nondenaturing gel. For DNase I footprints, reactions were treated with DNase I (Invitrogen) before resulting DNA fragments were run on a 6% denaturing gel. For both experiments, dried gels were exposed to a Biorad phosphorscreen, which was scanned using a Biorad Personal Molecular Imager.

#### In vitro transcription assays.

In vitro transcription experiments were carried out as previously described (76). Supercoiled pSR plasmid carrying the P*vca0224* DNA fragment was used as a template at a concentration of 16 μg/mL. The reaction buffer also contained 40 mM Tris-acetate pH 7.9, 5 mM MgCl_2_, 500 μM DTT, 50 mM KCl, 100 μg/mL BSA, 200 μM ATP/GTP/CTP, 10 μM UTP, and 5 μCi α^32^P-UTP. Reactions were incubated at 37 °C for 10 min with or without CRP and HapR as required (concentrations are detailed in figure legends). Where used, CRP was preincubated with 0.25 mM cAMP before addition to reactions. Reactions were started by adding 0.4 μM σ^70^-containing RNA polymerase and were incubated for 10 min at 37 °C. Transcripts were analyzed on a 6% denaturing polyacrylamide gel, which was imaged by exposure to a Biorad phosphorscreen and subsequent scanning using a Biorad Personal Molecular Imager.

#### RIL-seq experiment.

*V. cholerae* WT and *hfq::3XFLAG* strains were cultivated in triplicates in AKI medium. Cells were harvested after 1 h of aeration. The experiment was performed as previously described (45), with modifications in the rRNA depletion. Briefly, cells corresponding to 50 OD_600_ units were subjected to UV crosslinking, followed by cell lysis and coimmunoprecipitation using a monoclonal anti-FLAG antibody (Sigma; F1804). Coimmunoprecipitated RNA was trimmed with RNase A/T1 treatment, and RNA fragments were ligated using T4 RNA ligase. Following proteinase K digestion, RNA was extracted, fragmented, and treated with TurboDNase. rRNAs were depleted using custom-made biotinylated oligonucleotide probes as described previously (85). Oligos used for depletion are listed in *SI Appendix*, Table S3. rRNA-depleted RNA was purified using the Agencourt AMPure XP kit (Beckman Coulter Genomics) and used for cDNA library preparation. Libraries were amplified using NEBNext Ultra II Q5 polymerase and sequenced on a NextSeq1000 platform (Illumina) with 200-nt paired-end reads. Data analysis and visualization was performed according to our previously published computational pipeline (51). Chimeric reads were mapped to the *V. cholerae* reference genome (NCBI accession numbers NC_002505.1 and NC_002506.1).

## Supplementary Material

Appendix 01 (PDF)

## Data Availability

Raw reads and read count data of the RNA-Seq experiments were deposited into the Gene Expression Omnibus repository under accession number GSE311502 (86). Raw reads and RNA–RNA interaction data of the RIL-Seq experiments were deposited into the Gene Expression Omnibus repository under accession number GSE311501 (87). Documentation of the ChimericFragments is available at: https://github.com/maltesie/ChimericFragments (88).
