# Supplementary material for: A 3’UTR-derived small RNA modulates the life cycle of the cholera toxin–encoding filamentous phage, CTXϕ
Source: Proc Natl Acad Sci U S A. 2026 Jun 2;123(23):e2535142123. doi: 10.1073/pnas.2535142123 (PMC13250501; doi:10.1073/pnas.2535142123)
Supplement: Supplementary file 1 — Appendix 01 (PDF) [file pnas.2535142123.sapp.pdf]

Supplementary Information

A 3'UTR-derived small RNA modulates the life cycle of the cholera toxin-encoding filamentous phage, CTXφ

Anne Lippegaus[1], James R.J. Haycocks[2], Eoghan O'Driscoll[1], Marcel Sprenger[1], Kerstin Thriene[1,3], Elke-Martina Jung[1], Malte Siemers[1,3], Sebastian Krautwurst[1], David C. Grainger[2], Kai Papenfort[1,3]

1 Institute of Microbiology, General Microbiology, Friedrich Schiller University, 07743 Jena, Germany

2 Institute of Microbiology and Infection, School of Biosciences, University of Birmingham, Birmingham B15 2TT, UK

3 Microverse Cluster, Friedrich Schiller University, 07743 Jena, Germany

This supplement contains:

Figures S1-5

Tables S1-3

Supplemental References

TABLE OF CONTENTS

Figure S1 Expression of *ctxA* and *toxT* under virulence inducing conditions

Figure S2 RNase E-mediated processing of *cisR*

Figure S3 Transcriptional control of P*vca0224*

Figure S4 Interaction partners of selected CisR targets discovered by RIL-seq

Figure S5 Induction of CisR with MMC

Table S1 Strains used in this study

Table S2 Plasmids used in this study

Table S3 Oligonucleotides used in this study

Supplementary Figures

Figure S1.

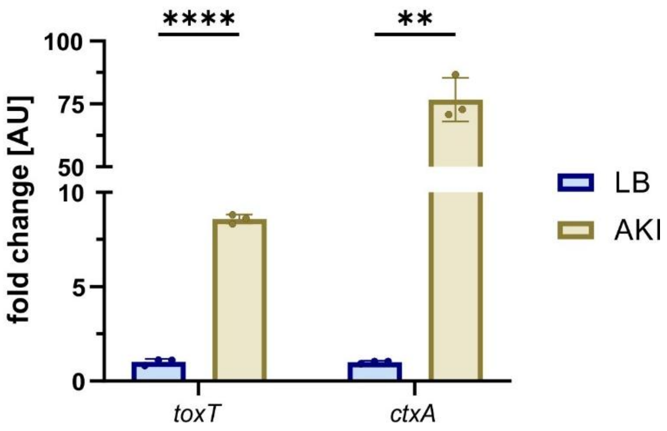

Figure S1: Expression of *ctxA* and *toxT* under virulence inducing conditions

ctxA and *toxT* mRNA levels are differentially expressed in virulence inducing conditions. RNA samples were obtained from *V. cholerae* wild-type cells were cultivated in LB and AKI medium. mRNA levels were determined by RT-qPCR. Data are presented as mean values of independent biological replicates ±SD, *n*=3. Statistical significance was calculated using an unpaired t test (∗∗$p \leq 0.01$, ∗∗∗∗$p \leq 0.0001$).

Figure S2.

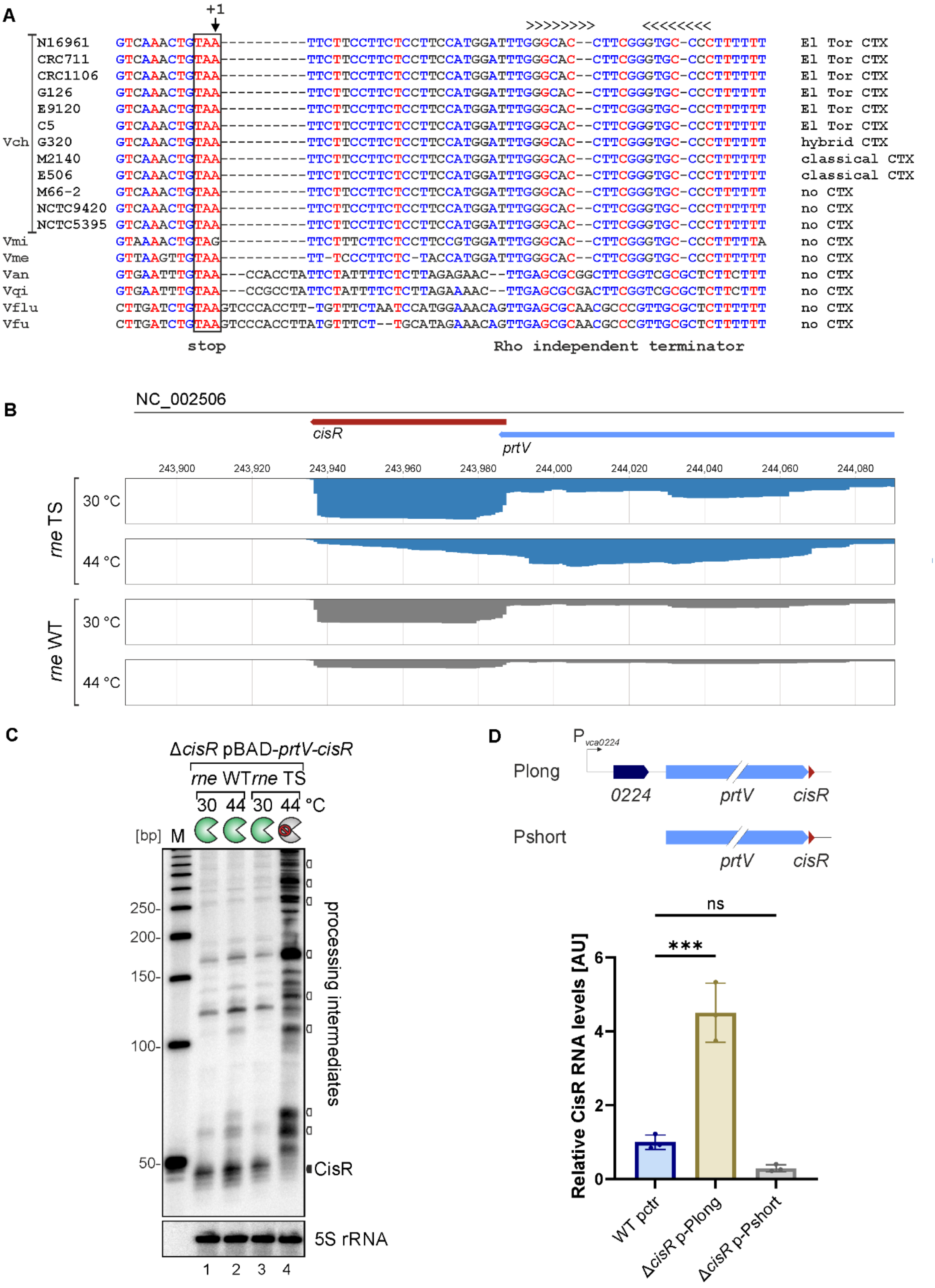
A
+1
>>>>>>>>
<<<<<<<<
Vch
N16961 GTCAAACTGTAA----------TTCTTCCTTCTCCTTCCATGGATTTGGGCAC--CTTCGGGTGC-CCCTTTTTT El Tor CTX
CRC711 GTCAAACTGTAA----------TTCTTCCTTCTCCTTCCATGGATTTGGGCAC--CTTCGGGTGC-CCCTTTTTT El Tor CTX
CRC1106 GTCAAACTGTAA----------TTCTTCCTTCTCCTTCCATGGATTTGGGCAC--CTTCGGGTGC-CCCTTTTTT El Tor CTX
G126 GTCAAACTGTAA----------TTCTTCCTTCTCCTTCCATGGATTTGGGCAC--CTTCGGGTGC-CCCTTTTTT El Tor CTX
E9120 GTCAAACTGTAA----------TTCTTCCTTCTCCTTCCATGGATTTGGGCAC--CTTCGGGTGC-CCCTTTTTT El Tor CTX
C5 GTCAAACTGTAA----------TTCTTCCTTCTCCTTCCATGGATTTGGGCAC--CTTCGGGTGC-CCCTTTTTT El Tor CTX
G320 GTCAAACTGTAA----------TTCTTCCTTCTCCTTCCATGGATTTGGGCAC--CTTCGGGTGC-CCCTTTTTT hybrid CTX
M2140 GTCAAACTGTAA----------TTCTTCCTTCTCCTTCCATGGATTTGGGCAC--CTTCGGGTGC-CCCTTTTTT classical CTX
E506 GTCAAACTGTAA----------TTCTTCCTTCTCCTTCCATGGATTTGGGCAC--CTTCGGGTGC-CCCTTTTTT classical CTX
M66-2 GTCAAACTGTAA----------TTCTTCCTTCTCCTTCCATGGATTTGGGCAC--CTTCGGGTGC-CCCTTTTTT no CTX
NCTC9420 GTCAAACTGTAA----------TTCTTCCTTCTCCTTCCATGGATTTGGGCAC--CTTCGGGTGC-CCCTTTTTT no CTX
NCTC5395 GTCAAACTGTAA----------TTCTTCCTTCTCCTTCCATGGATTTGGGCAC--CTTCGGGTGC-CCCTTTTTT no CTX
Vmi GTAAAACTGTAG----------TTCTTTCTTCTCCTTCCGTGGATTTGGGCAC--CTTCGGGTGC-CCCTTTTTA no CTX
Vme GTTAAGTTGTAA----------TT-TCCCTTCTC-TACCATGGATTTGGGCAC--CTTCGGGTGC-CCCTTTTTT no CTX
Van GTGAATTTGTAA---CCACCTATTCTATTTTCTCTTAGAGAAC--TTGAGCGCGGCTTCGGTCGCGCTCTTCTTT no CTX
Vqi GTGAATTTGTAA---CCGCCTATTCTATTTTCTCTTAGAAAAC--TTGAGCGCGACTTCGGTCGCGCTCTTCTTT no CTX
Vflu CTTGATCTGTAAGTCCCACCTT-TGTTTCTAATCCATGGAAACAGTTGAGCGCAACGCCCGTTGCGCTCTTTTTT no CTX
Vfu CTTGATCTGTAAGTCCCACCTTATGTTTCT--TGCATAGAAACAGTTGAGCGCAACGCCCGTTGCGCTCTTTTTT no CTX
stop
Rho independent terminator
B
NC_002506
cisR
prtV
243,900
243,920
243,940
243,960
243,980
244,000
244,020
244,040
244,060
244,080
rne TS
30 °C
44 °C
rne WT
30 °C
44 °C
C
ΔcisR pBAD-prtV-cisR
rne WT
rne TS
30 44 30 44 °C
[bp] M
250-
200-
150-
100-
50-
processing intermediates
CisR
5S rRNA
1 2 3 4
D
P vca0224
Plong
0224
prtV
cisR
Pshort
prtV
cisR
ns
***
Relative CisR RNA levels [AU]
0
2
4
6
WT pctr
ΔcisR p-Plong
ΔcisR p-Pshort

Figure S2: RNase E-mediated processing of *cisR*

(A) Sequence alignment of *cisR* homologues in different *Vibrio cholerae* strains used by Hu et al (1) and closely related *Vibrio* species. The *cisR* sequences were aligned using the Multalin tool (2). The stop codon of *prtV* is boxed and the Rho-independent terminator are indicated. The right site indicates the biotype variant of CTXφ. *Vch, Vibrio cholerae; Vmi, Vibrio mimicus; Vme, Vibrio metoecus; Van, Vibrio anguillarum; Vqi, Vibrio qinghaiensis; Vflu, Vibrio fluvialis; Vfu, Vibrio furnissii.*
(B) Read-mappings of TIER-seq (3) to the *cisR* locus. The y-axes were set to the same scale. The positions in *V. cholerae* C6706 genome are indicated above the sequencing tracks. *prtV* and *cisR* are annotated. The reads for *rne* WT are presented in grey and for *rne* TS in blue. Temperatures are indicated on the left side of the tracks.
(C) Influence of RNase E on CisR levels. *V. cholerae* Δ*cisR* carrying either a wild-type (*rne* WT) or temperature-sensitive RNase E (*rne* TS) allele and the pBAD-*prtV-cisR* vector were cultivated at 30°C in LB medium. AT OD_{600} of 1.0, cultures were split and kept at 30°C or shifted to 44°C for 30min. Expression of *prtV-cisR* was induced using L-arabinose (0.2%, 30min) and CisR levels were monitored by northern blot. Probing for 5S ribosomal RNA served as loading control.
(D) Promotor identification of *cisR*. Quantification northern Blot, Promotor identification. For statistical analysis, an ordinary one-way ANOVA with Dunnet's multiple comparison test was performed (ns, not significant, $***p \leq 0.001$).

Figure S3.

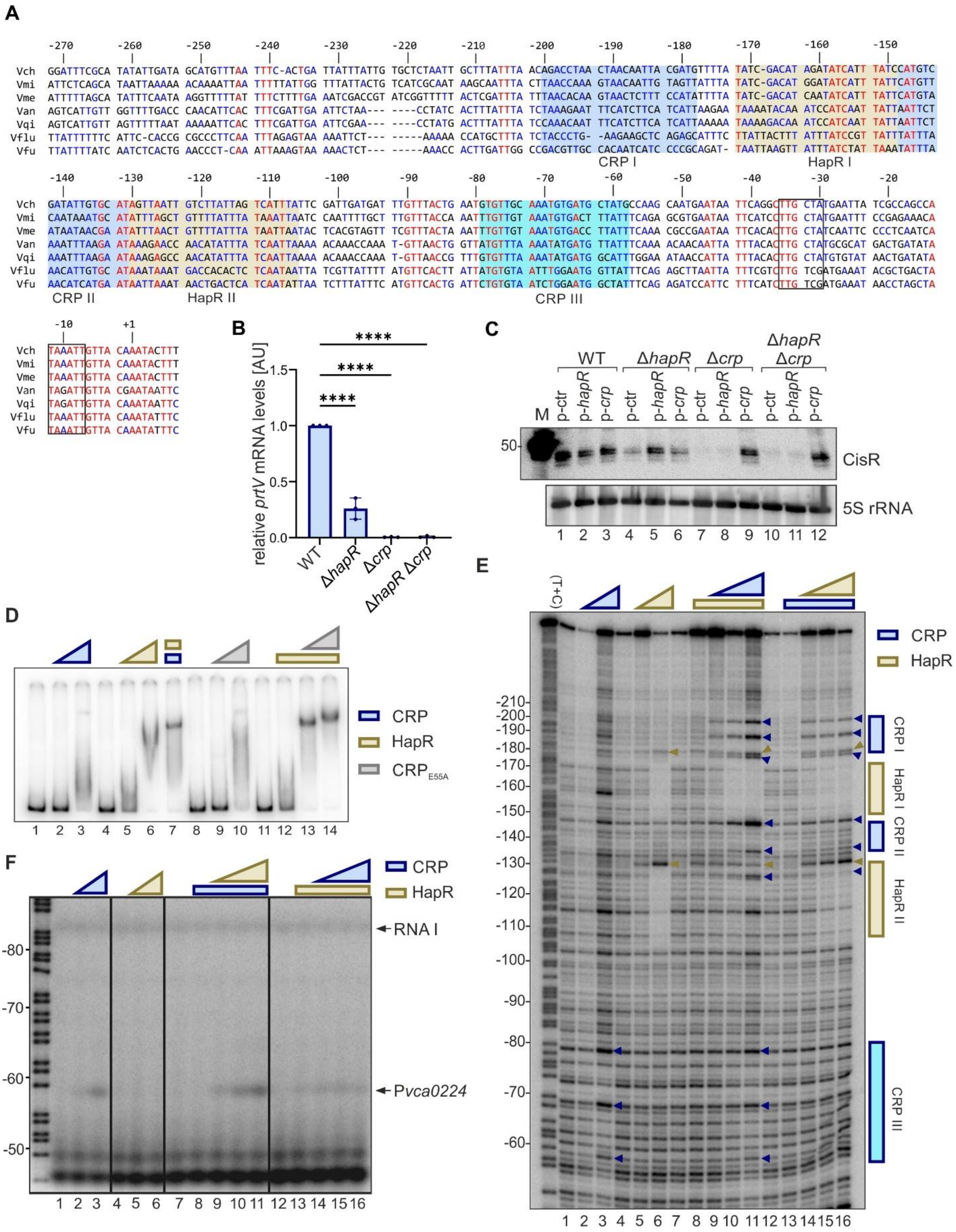
A
CRP I
HapR I
CRP II
HapR II
CRP III
B
relative prtV mRNA levels [AU]
WT
ΔhapR
Δcrp
ΔhapR Δcrp
C
WT
ΔhapR
Δcrp
ΔhapR Δcrp
M
p-ctr
p-hapR
p-crp
CisR
5S rRNA
D
CRP
HapR
CRP E55A
E
(T+C)
CRP
HapR
F
CRP
HapR
RNA I
Pvca0224

Figure S3: Transcriptional control of P*vca0224*

(A) Alignment of *cisR* promoter sequence from various *Vibrio* species. The -35 box, -10 box and TSS (+1) are indicated. Putative HapR and CRP binding sites are highlighted in gold and blue, respectively. An additional (weaker) CRP binding site is indicated in light blue. *Vch, Vibrio cholerae; Vmi, Vibrio mimicus; Vme, Vibrio metoecus; Van, Vibrio anguillarum; Vqi, Vibrio qinghaiensis; Vflu, Vibrio fluvialis; Vfu, Vibrio furnissii.*

(B) Role of HapR and CRP for *prtV* levels. *V. cholerae* wild-type, Δ*hapR*, Δ*crp* and Δ*hapR* Δ*crp* cells were cultivated in LB medium and RNA samples were collected at OD_{600} of 1.0. *prtV* mRNA levels were determined by qRT-PCR. For statistical analysis, an ordinary one-way ANOVA with Dunnetts's multiple comparisons test was used (∗∗∗∗$p \leq 0.0001$).

(C) Role of HapR and CRP for CisR levels. *V. cholerae* wild-type, Δ*hapR*, Δ*crp* and Δ*hapR* Δ*crp* cells harboring either an empty control vector (p-ctr), a *hapR* overexpression plasmid (p-*hapR*) or a *crp* overexpression plasmid (p-*crp*) were cultivated in LB medium and RNA samples were collected at OD_{600} of 1.0. Northern blot analysis was performed to determine CisR levels and probing for 5S ribosomal RNA served as loading control.

(D) Electrophoretic mobility shift assay of CRP_{E55A} binding to P*vca0224*. Radiolabeled P*vca0224* DNA fragment was incubated alone (lanes 1, 4, 8 and 11), with increasing concentrations from 0.5 µM to 2 µM of purified CRP (lanes 2-3), HapR (lanes 5-6) and CRP_{E55A} (lanes 9-10). In lane 7 the P*vca0224* fragment was incubated with 0.5 µM HapR and CRP respectively and in lanes 12-14 with constant concentrations of HapR (0.5 µM) while increasing the CRP_{E55A} concentration from 0 µM to 2 µM. Complexes were separated on a native polyacrylamide gel and visualized by autoradiography.

(E) Binding locations of HapR and CRP upstream of *vca0224* were determined by DNase I footprinting. The pattern of DNase I cleavage in the absence of any proteins is shown in lanes 1, 4, 7 and 12. Radiolabeled P*vca0224* DNA fragment was incubated with increasing concentrations of CRP (lanes 2-3) or HapR (lanes 5-6) respectively, from 0.5-2 µM. In lanes 8-11 the P*vca0224* fragment was incubated with constant concentrations of HapR (0.5 µM) while CRP concentrations were increased (from 0 µM to 2 µM) and vice versa in lanes 13-16. Changes in the cleavage pattern due to the presence of CRP and/ or HapR are indicated at the right side of the gel by blue (light blue) and golden boxes, respectively, and are mapped onto the sequence shown in Figure S3A. Colored arrows indicate DNase I hypersensitive bands that appear only in the presence of CRP (blue) or HapR (gold). The gel is calibrated with a Maxam-Gilbert ladder, numbering shows the position relative to the transcription start site.

(F) *In vitro* transcription experiment showing the effects of HapR and CRP on transcription from P*vca0224*. The P*vca0224* fragment was cloned *Eco*RI-*Hin*dIII into plasmid pSR. The resulting construct was incubated with *V. cholerae* RNA polymerase in the absence of CRP or HapR (lanes 1, 4, 7, and 12), or was incubated with increasing concentrations of CRP (lanes 2-3) or HapR (lanes 5-6) respectively, from 0.5-2 µM. In lanes 8-11 the plasmid construct was incubated with *V. cholerae* RNA polymerase, HapR concentrations were kept constant (0.5 µM), while CRP concentrations were increased (up 0 µM to 2 µM) and *vice versa* in lanes 13-16 with the same concentrations. Black arrows indicate transcripts derived from P*vca0224* and the plasmid RNA transcript I used as an internal control. The gel is calibrated with a Maxam-Gilbert ladder. Numbering refers to transcript size in nucleotides.

Figure S4.

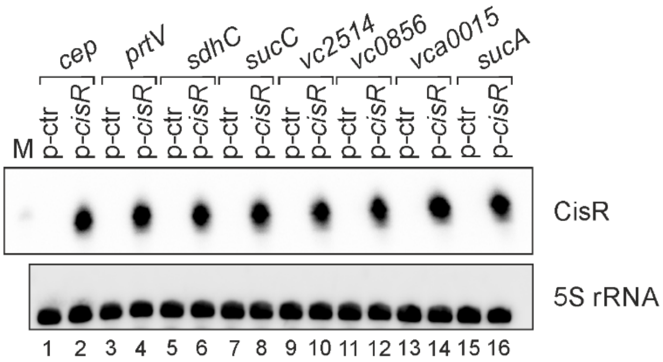

B

```
                  prtV
          -22          -17
             | C    |
5'-ACU..UAAAU  ↑     AUAUU..GGC-3'
              AAGGAA
              ||||||
              UUCCUU
3'-UUU..UCCUC  ↓     CUU -5'
             | G    |
           +10     +5
                 CisR
```

C

```
                           sdhC
            +11                              +33
             |                                |
5'-GUC..CAAGC      AA   AA     AGUC     CCUGU..AUU-3'
             CCGUG   AG   AGAA     AAGA
             |||||   ||   ||||     ||||
             GGUAC   UC   UCUU     UUCU
3'-UUU..GUUUA      CU   C      CC       UA-5'
             |                                |
            +22                              +3
                           CisR
```

D

```
                     sucC
              -35        -26
               |          |
5'-CUC..UGGAC               AACAA..CCG-3'
              UCUAUGGAAG
              ||||||||||
              AGGUACCUUC
3'-UUU..GGUUU               CUCUU..UUA-5'
               |          |
              +23        +14
                     CisR
```

E

```
                      vc0856
            -18                   -2
             |                     |
5'-GAA..GUAAU      G    ACU       CAUGU..GAU-3'
             AAAUU  GUG     GAGAA
             |||||  |||     |||||
             UUUAG  UAC     CUCUU
3'-UUU..ACGGG      G    CUUC      CCUUCUUA-5'
             |                     |
            +26                   +9
                      CisR
```

F

```
                     vc2514
              -3          +7
               |          |
5'-GCC..AGGUU              AAGUU..UCA-3'
              UUUAUGGAA
              |||||||||
              AGGUACCUU
3'-UUU..GGUUU              CCUCU..UUA-5'
               |          |
              +23        +15
                     CisR
```

G

```
                  vca0015
            -37       -29
             |         |
5'-CAC..AAGCC           UAUUC..GCA-3'
             GGAAGGGGA
             |||||||||
             CCUUCCUCU
3'-UUU..AGGUA           UCCUUCUUA-5'
             |         |
            +18       +10
                 CisR
```

H

```
                      sucA
              -15         -6
               |           |
5'-GCA..CUACU  UU           AAAAA..GCA-3'
              GG   AAGGGA
              ||   ||||||
              CC   UUCCUU
3'-UUU..ACCUU  UC           CUUA-5'
               |           |
              +14         +5
                      CisR
```

I

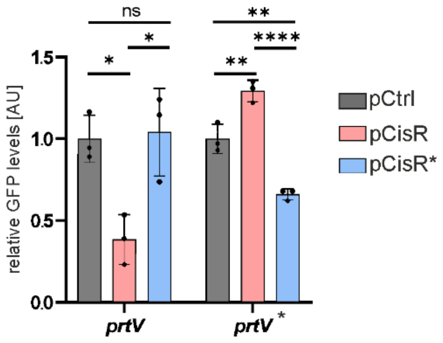

Figure S4: Interaction partners of selected CisR targets discovered by RIL-seq

(A) Plasmid-borne expression of *cisR* in *E. coli* Top10 cells. RNA samples are corresponding to Fig. 3C. CisR levels were determined by northern blotting and probing for 5S ribosomal RNA served as loading control.
(B)-(H) Predicted base-pairing regions between CisR and its targets validated in Fig. 3C by ChimericFragments (4) and IntaRNA (5): *prtV* (B), *sdhC* (C), *sucC* (D), *vc0856* (E), *vc2514* (F), *vca0015* (G) and *sucA* (H). Start codons are underlined.
(I) Compensatory mutation CisR and *prtV*. Translational GFP reporter fusion of *prtV*/ *prtV** were co-transformed with constitutive CisR/ CisR* expression plasmids or an empty control plasmid in *E. coli* Top10 cells. The mutations in *prtV** and CisR* are highlighted in (B). GFP production was measured, fluorophore levels from the control strains were set to 1 and the fold repression was plotted. For statistical analyses, a two-way ANOVA with Dunnett's multiple comparison test was used (ns, not significant, ∗$p \leq 0.05$, ∗∗$p \leq 0.01$, ∗∗∗∗$p \leq 0.0001$).

Figure S5.

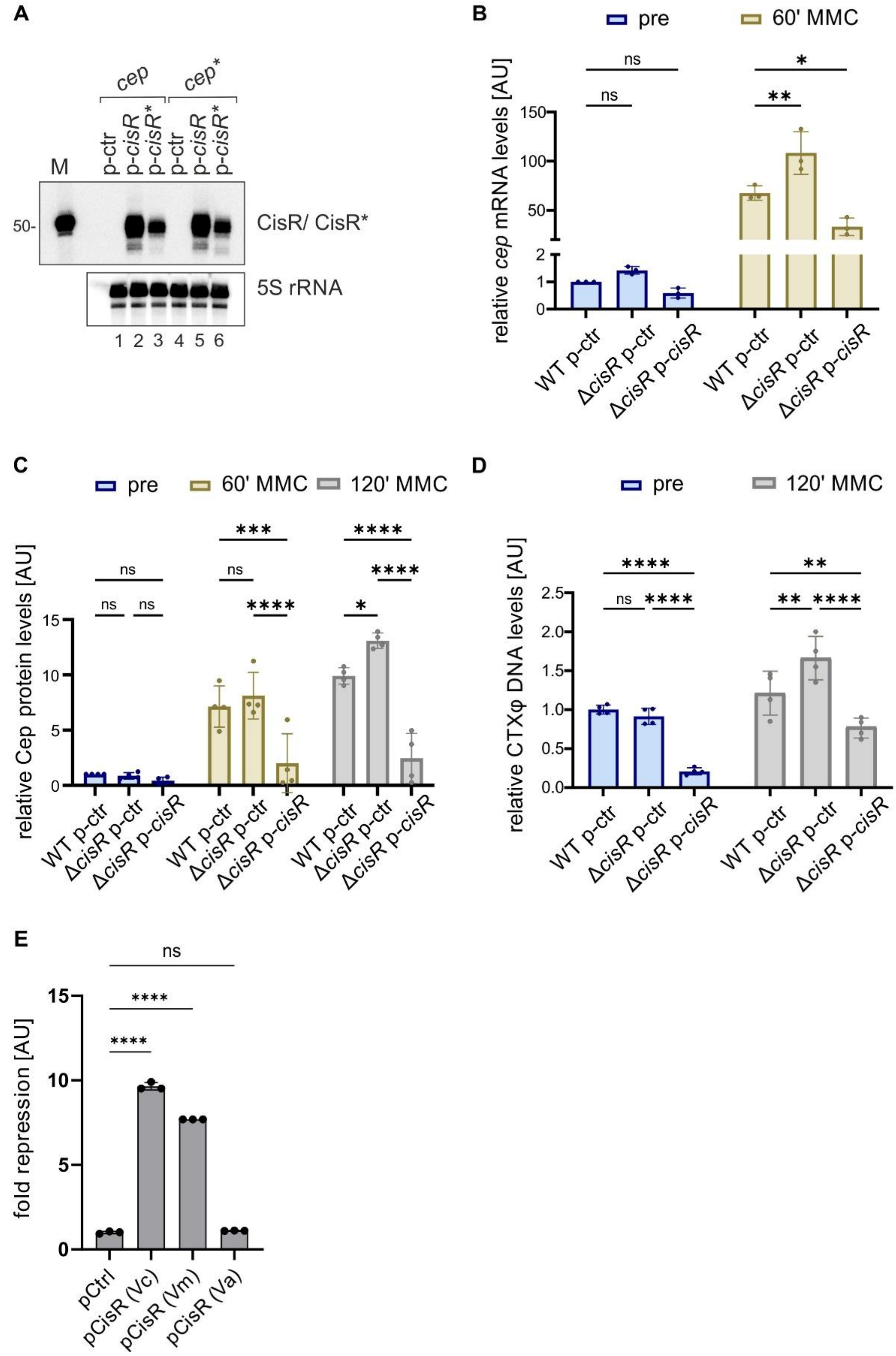

A
cep
cep*
M
p-ctr
p-cisR
p-cisR*
p-ctr
p-cisR
p-cisR*
50-
CisR/ CisR*
5S rRNA
1 2 3 4 5 6
B
pre
60' MMC
ns
ns
*
**
relative cep mRNA levels [AU]
WT p-ctr
ΔcisR p-ctr
ΔcisR p-cisR
WT p-ctr
ΔcisR p-ctr
ΔcisR p-cisR
C
pre
60' MMC
120' MMC
relative Cep protein levels [AU]
ns
***
****
****
****
*
WT p-ctr
ΔcisR p-ctr
ΔcisR p-cisR
D
pre
120' MMC
****
**
ns
relative CTXφ DNA levels [AU]
WT p-ctr
ΔcisR p-ctr
ΔcisR p-cisR
E
ns
****
fold repression [AU]
pCtrl
pCisR (Vc)
pCisR (Vm)
pCisR (Va)

Figure S5: Induction of CisR with MMC

(A) Plasmid-borne expression of *cisR* and *cisR** in *E. coli* Top10 cells. RNA samples correspond to Fig. 4B. CisR/ CisR* levels were determined by northern blotting and probing for 5S ribosomal RNA served as loading control.
(B-D) Effect of MMC on *cep* mRNA (B), Cep protein levels (C) and extracellular CTXφ DNA (D). **(B-C)** RNA (B) and protein samples (C) of *V. cholerae cep::3XFLAG* p-ctr, Δ*cisR cep::3XFLAG* p-ctr and Δ*cisR cep::3XFLAG* p-cisR were collected at indicated timepoints post treatment with MMC (200 ng/mL). *cep* mRNA levels are determined by qRT-PCR. Protein levels of Cep::3XFLAG were monitored by western blotting RNAP served as loading controls for western blot. **(D)** Extracellular CTXϕ DNA levels were measured in cell-free and DNase-treated supernatants of *V. cholerae* wild-type p-ctr, Δ*cisR* p-ctr and Δ*cisR* p-cisR using qPCR. DNA levels were quantified before and 60 and 120 minutes post treatment with MMC (200 ng/mL). The levels of CTXϕ DNA before MMC treatment in the wild-type p-ctr were set to 1 and the relative production of extracellular CTXϕ phage DNA was plotted. For statistical analysis, a two-way ANOVA with Tukey's multiple comparison test was used (ns, not significant, $*p \leq 0.05$, $**p \leq 0.01$, $***p \leq 0.001$, $****p \leq 0.0001$). **(E)** Validation of CisR targets predicted by RIL-seq. Translational GFP reporter fusions were co-transformed with a constitutive CisR expression plasmid (with CisR from *V. cholerae* (Vc*), V. mimicus* (Vm) or *V. anguillarum* (Va)) or an empty control plasmid in *E. coli* Top10 cells. GFP production was measured, fluorophore levels from the control strains were set to 1 and the fold repression was plotted. Error bars represent the SD of three independent biological replicates. For statistical analyses, a one-way ANOVA with Dunnett's multiple comparison test was used (ns, not significant, $****p \leq 0.0001$).

Table S1: Strains used in this study

Strain	Relevant markers / Genotype	Reference / Source
V. cholerae		
KPS-0014	C6706 wild-type	(6)
KPS-0053	C6706 Δ*hapR*	(7)
KPS-0995	C6706 *hfq::3XFLAG*	(8)
KPVC-10966	C6706 Δ*hapR* Δ*crp*	This study
KPVC-10984	C6706 Δ*crp*	(9)
KPVC-12925	C6706 *hapR::3XFLAG*	(10)
KPVC-13484	C6706 *crp::3XFLAG*	This study
KPVC-14998	C6706 Δ*cisR*	This study
KPVC-10141	C6706 *rne*-3071	(3)
KPVC-15015	C6706 *rne*-3071 Δ*cisR*	This study
KPVC-15080	C6706 *cep::3XFLAG*	This study
KPVC-15081	C6706 Δ*cisR cep::3XFLAG*	This study
KPVC-15469	C6706 Δ*hapR crp::3XFLAG*	This study
KPVC-15538	C6706 Δ*crp hapR::3XFLAG*	This study
KPVC-16325	C6706 Δ*cisR*::*CisR**	This study
KPVC-16337	C6706 Δ*prtV*	This study
E. coli		
Top10	*F- mcrA Δ(mrr-hsdRMS-mcrBC) φ80lacZΔM15 ΔlacX74 nupG recA1 araD139 Δ(ara-leu)7697 galE15 galK16 rpsL(StrR) endA1 λ-*	Invitrogen
S17λpir	*ΔlacU169 (ΦlacZΔM15), recA1, endA1, hsdR17, thi-1, gyrA96, relA1, λpir*	New England Biolabs

Table S2: Plasmids used in this study

Plasmid trivial name	Plasmid Stock Name	Relevant fragment	Comment	Origin, marker	Reference
pXG10-sfGFP	pXG10-sfGFP	lacZ'::sfGFP	Template plasmid for translational reporter	pSC101*, Cm^R	(11)
pXG10-*vc1461* (*cep*)	pAL42	5'UTR + 20 aa of *vc1461*	Translational GFP reporter	pSC101*, Cm^R	This study
pXG10-*vc1461* (*cep*)*	pAL84	5'UTR + 20 aa of *vc1461**	Translational GFP reporter	pSC101*, Cm^R	This study
pXG10-*vc2087* (*sucA*)	pAL72	5'UTR + 20 aa of *vc2087* (*sucA)*	Translational GFP reporter	pSC101*, Cm^R	This study
pXG10-*vca0015*	pAL76	5'UTR + 20 aa of *vc0015*	Translational GFP reporter	pSC101*, Cm^R	This study
pXG10-*vc0856*	pAL75	5'UTR + 20 aa of *vc0856*	Translational GFP reporter	pSC101*, Cm^R	This study
pXG10-*vc2091* (*sdhC*)	pSM003	5'UTR + 20 aa of *vc2091* (*sdhC*)	Translational GFP reporter	pSC101*, Cm^R	This study
pXG10-*vc2085* (*sucC*)	pSM002	5'UTR + 20 aa of *vc2085*	Translational GFP reporter	pSC101*, Cm^R	(4)
pXG10-*vc2514*	pAL73	5'UTR + 20 aa of *vc2514*	Translational GFP reporter	pSC101*, Cm^R	This study
pXG10-*vca0223* (*prtV*)	pNP83	5'UTR + 15 aa of *prtV*	Translational GFP reporter	pSC101*, Cm^R	This study
pCMW-1	pCMW-1		Control plasmid	p15A, Kan^R	(12)
p-*cisR*	pAL41	*cisR*	*cisR* expression plasmid	p15A, Kan^R	This study
p-*cisR* C8G G21C G22C*	pAL85	*cisR* C8G G21C G22C*	*cisR* C8G G21C G22C* expression plasmid	p15A, Kan^R	This study
pEVS143	pEVS143	P*tac* promotor	Constitutive expression plasmid (template)	p15A, Kan^R	(13)
pEVS-protein	pMD80	P*tac* promotor, 5'UTR, MCS, and T1 terminator	Protein expression plasmid	p15A, Kan^R	(9)
p-*vc0583 (hapR*)	pDD11	*vc0583 (hapR*)	*vc0583 (hapR*) expression plasmid	p15A, Kan^R	This study
p-*vc2614 (crp)*	pAL79	*vc2614 (crp)*	*vc2614 (crp)* expression plasmid	p15A, Kan^R	This study
pBAD1K-ctr	pMD004		Control plasmid	p15A, Kan^R	(3)
pBAD1K-*prtV-cisR*	pAL50	*prtV-cisR*	Inducible *prtV-cisR* expression plasmid	p15A, Kan^R	This study
p-P*vca0224-prtV-cisR* (P_{long})	pAL59	P*vca0224-prtV-cisR*	P*vca0224-prtV-cisR* expression plasmid	p15A, Kan^R	This study
p-*prtV-cisR* (P_{short})	pAL60	*prtV-cisR*	*prtV-cisR* expression plasmid	p15A, Kan^R	This study
p-P*vca0224::GFP*	pAL82	P*vca0224::GFP*	Transcriptional reporter for CisR	p15A, Kan^R	This study
pKAS32	pKAS32		suicide plasmid for allelic exchange	R6K, Amp^R	(14)
pKAS32-Δ*cisR*	pAL48	up-/downstream flanks of *cisR*	suicide plasmid for *cisR* knock-out	R6K, Amp^R	This study
pKAS32-*vc1461(cep)::3XFLAG*	pAL55	*vc1461(cep)::3XFLAG*	*vc1461(cep)::3XFLAG* allelic replacement	R6K, Amp^R	This study

pKAS32-*vc2614(crp)::3XFLAG*	pKV126	*vc2614(crp)::3XFLAG*	*vc2614(crp)::3XFLAG* allelic replacement	R6K, Amp^R	This study
pBAD-1C-native-5'UTR-rstC	pAL89	*rstC*	Inducible *rstC* expression plasmid	P15A, Cm^R	This study
pKAS32- *vc0583 (hapR)::3XFLAG*	pASp017	*vc0583 (hapR)::3XFLAG*	*vc0583 (hapR)::3XFLAG* allelic replacement	R6K, Amp^R	(10)
pKAS32-Δ*vc2614(crp)*	pRH023	up-/downstream flanks of *crp*	suicide plasmid for *crp* knock-out	R6K, Amp^R	(9)
pKAS32-*cisR**	pMS382	up-/downstream flanks of *cisR*	suicide plasmid for introducing *cisR* C8G G21C G22C*	R6K, Amp^R	This study
pKAS32-Δ*prtV*	pMS383	up-/downstream flanks of *prtV*	suicide plasmid for *prtV* knock-out	R6K, Amp^R	This study
pXG10-*prtV**	pMS384	5'UTR + 15 aa of *prtV*	Translational GFP reporter	pSC101*, Cm^R	This study
p-*cisR* M4*	pAL52	*cisR C8G*	*cisR* M4 *C8G* expression plasmid	p15A, Kan^R	This study
p-*cisR* (*V. mimicus*)	pMS390	*cisR* (*V. mimicus*)	*cisR* (*Vm*) expression plasmid	p15A, Kan^R	This study
p-*cisR* (*V. anguillarium*)	pMS391	*cisR* (*V. anguillarium*)	*cisR* (*Va*) expression plasmid	p15A, Kan^R	This study
pSR	pSR	P*vca0224*	*In vitro* transcription	pMB1 Amp^R	(15)

Table S3: Oligonucleotides used in this study

Name	Sequence 5' to 3'	Description
**Oligonucleotides for plasmid construction**		
KPO-00092	CCACACATTATACGAGCCGA	Plasmid construction (pEVS143)
KPO-00196	GGAGAAACAGTAGAGAGTTGCG	Plasmid construction (pBAD)
KPO-00267	TAATAGGCCTAGGATGCATATG	Plasmid construction (pKAS32)
KPO-00268	CGTTAACAACCGGTACCTCTA	Plasmid construction (pKAS32)
KPO-01397	GATCCGGTGATTGATTGAGC	Plasmid construction (pEVS143, pBAD)
KPO-01423	TCTAGATTAAATCAGAACGCAGAAG	Plasmid construction (pBAD)
KPO-01702	ATGCATGTGCTCAGTATCTCTATC	Plasmid construction (pXG10)
KPO-01703	GCTAGCGGATCCGCTGG	Plasmid construction (pXG10)
KPO-01952	AGGCCTAGTTAAGGAGATATACA	Plasmid construction (pCMW)
KPO-01953	GTCGACAATGAAGGGTCTTTTA	Plasmid construction (pCMW)
KPO-02757	TGAGGATCCGGTGATTGATTGAGCA	Plasmid construction (pCMW)
KPO-02758	AATGAAGGGTCTTTTATGATCTTAT	Plasmid construction (pDD11)
KPO-02759	AAAAGACCCTTCATTCTAGTTCTTATAGATACACA	Plasmid construction (pDD11)
KPO-02760	ATCACCGGATCCTCAATCCTCGCTTTGTTAT	Plasmid construction (pDD11)
KPO-03362	GTTTTTATGCATAAATACTTTACATATGGATATGTACTATG	Plasmid construction (pNP83)
KPO-03363	GTTTTTGCTAGCGCCTAAATCAATGGGTGTTTGAG	Plasmid construction (pNP83)
KPO-04168	GACTACAAAGACCATGACGG	Plasmid construction (pAL55)
KPO-05249	GAGATACTGAGCACATGCATGTCGCCGATTTGGCGGTTGAAATG	Plasmid construction (pSM003)

KPO-05250	CCAGCGGATCCGCTAGCAATGGTCTGCAAATCTAAATTAAC	Plasmid construction (pSM003)
KPO-05416	TTACTATTTATCGTCATCTTTGTAGTCG	Plasmid construction (pAL55)
KPO-06040	TAGAGGTACCGGTTGTTAACGGGATGAGAGTTTTGTGGTGATC	Plasmid construction (pKV126)
KPO-06041	GCGAGTGCCGTAAACCACG	Plasmid construction (pKV126)
KPO-06042	CGTGGTTTACGGCACTCGCGACTACAAAGACCATGACGGT G	Plasmid construction (pKV126)
KPO-06043	GACGGGTTATCGGGGCACCTATTTATCGTCATCTTTGTAGTCG	Plasmid construction (pKV126)
KPO-06044	GTGCCCCGATAACCCGTC	Plasmid construction (pKV126)
KPO-06045	CATATGCATCCTAGGCCTATTACAACGCTGCTTCCTGCTACC	Plasmid construction (pKV126)
KPO-08333	TCGGCTCGTATAATGTGTGGATTCTTCCTTCTCCTTCCATG	Plasmid construction (pAL41)
KPO-08334	GCTCAATCAATCACCGGATCAAAAAAGGGGCACCCGAAGGTGC	Plasmid construction (pAL41)
KPO-08335	GAGATACTGAGCACATGCATCATCCTTTGGGATTGGCGC	Plasmid construction (pAL42)
KPO-08336	GAGCCAGCGGATCCGCTAGCAACCCCGAGTGAAAGCGTG	Plasmid construction (pAL42)
KPO-08384	CGCAACTCTCTACTGTTTCTCCATGAAAACGATCAAAAAAACGCT ATTA	Plasmid construction (pAL50)
KPO-08385	CTCAATCAATCACCGGATC GGCTTTTCGCATTGGCATGA	Plasmid construction (pAL50)
KPO-08386	AGAGGTACCGGTTGTTAACGTGCTTGGTATTCACTCCCTG	Plasmid construction (pAL48)
KPO-08387	ATCACCATCAAAGTCAAACTGTAA GGGCACCTTCGGGTGC	Plasmid construction (pAL48)
KPO-08388	TTACAGTTTGACTTTGATGGTGAT	Plasmid construction (pAL48)
KPO-08389	TATGCATCCTAGGCCTATTA TTCCACGAAGCCAATCACTATTA	Plasmid construction (pAL48)
KPO-08418	TAGAGGTACCGGTTGTTAACGTGCGCGTACTCGGCCTC	Plasmid construction (pAL55)
KPO-08419	CCGTCATGGTCTTTGTAGTCTTTAGCCTTACGAATTAAGCCAAT	Plasmid construction (pAL55)

KPO-08420	CGACTACAAAGATGACGATAAATAGTAATAGTGCTTGAGTTGTG GCTG	Plasmid construction (pAL55)
KPO-08421	CATATGCATCCTAGGCCTATTACGCCGTGTTCATGGTGTC	Plasmid construction (pAL55)
KPO-08766	TCTTCGTTCTCCTTCCATGGATTTGGG	Plasmid construction (pAL52)
KPO-08767	GGAGAACGAAGAATCCACACATTATACGAG	Plasmid construction (pAL85, pAL52)
KPO-08924	TAAAAGACCCTTCATTGTCGACACAGACCTAACTAACAATTACGAT	Plasmid construction (pAL59)
KPO-08925	TAAAAGACCCTTCATTGTCGACATGAAAACGATCAAAAAAACGCT ATT	Plasmid construction (pAL60)
KPO-08926	TGCTCAATCAATCACCGGATCCTCAAAAAAAGGGGCACCCGAAG	Plasmid construction (pAL59, pAL60)
KPO-09376	GAGATACTGAGCACATGCATGCAGGCCTTCGGGCC	Plasmid construction (pAL72)
KPO-09377	GAGCCAGCGGATCCGCTAGCTGCATTGGCGCCAGCCA	Plasmid construction (pAL72)
KPO-09378	GAGATACTGAGCACATGCATGCCGCGTGAAGAAACAGC	Plasmid construction (pAL73)
KPO-09379	GAGCCAGCGGATCCGCTAGCTGAAATGGTCACTTCGCCTTG	Plasmid construction (pAL73)
KPO-09382	GAGATACTGAGCACATGCATGAACTTTGAAGCAGCGGGC	Plasmid construction (pAL75)
KPO-09383	GAGCCAGCGGATCCGCTAGCATCACGCTCGGAGGCGT	Plasmid construction (pAL75)
KPO-09384	GAGATACTGAGCACATGCATCACGCTTTGCCCGCGC	Plasmid construction (pAL76)
KPO-09385	GAGCCAGCGGATCCGCTAGCTGCACGCGCCAACTGATC	Plasmid construction (pAL76)
KPO-09649	GCTAACAGGAGGAATTAACCATGGTTCTAGGTAAACCTCAAAC	Plasmid construction (pAL79)
KPO-09650	TCGTTTTATTTGATGCCTCTAGATTATTAGCGAGTGCCGTAAACCA	Plasmid construction (pAL79)
KPO-09800	AAAGACCCTTCATTGTCGACTATTGATAGCATGTTTAATTTCACTG	Plasmid construction (pAL82)
KPO-09801	ATATCTCCTTAACTAGGCCTGTAACAATTTATGGCTGGCGA	Plasmid construction (pAL82)

KPO-10106	GGAGAACCTACATGTTTAGCTC	Plasmid construction (pAL84)
KPO-10107	CATGTAGGTTCTCCTTTACCGT	Plasmid construction (pAL84)
KPO-10158	TCTTCGTTCTCCTTCCATCCATTTGGG	Plasmid construction (pAL85)
KPO-10633	CGCAACTCTCTACTGTTTCTCCTAGAGCTCATGCCATATTGAATT	Plasmid construction (pAL89)
KPO-10634	CTTCTGCGTTCTGATTTAATCTAGATTACAGTGATGGCTCAGTCAAT	Plasmid construction (pAL89)
KPO-12115	AGAGGTACCGGTTGTTAACGCAACGGTCTGCTTGGTATTC	Plasmid construction (pMS382)
KPO-12116	TATGCATCCTAGGCCTATTAGCTTTGCGTTCCAAGGTTTC	Plasmid construction (pMS382)
KPO-12117	ATTTGGGCACCTTCGGGTG	Plasmid construction (pMS382)
KPO-12123	GCACCCGAAGGTGCCCAAATGGATGGAAGGAGAACGAAGAATTACAGT TTGACTTTG	Plasmid construction (pMS382)
KPO-12159	AGAGGTACCGGTTGTTAACG GGTCTGCTTGGTATTCACTCC	Plasmid construction (pMS383)
KPO-12160	GCGGCGCAGTCACCACC	Plasmid construction (pMS383)
KPO-12161	GATGGTGGTGACTGCGCCGCTTTATTTCCTTAATATTTCCTTATTTAAG	Plasmid construction (pMS383)
KPO-12162	TATGCATCCTAGGCCTATTA TGTACCAACCTAGGCCAAAC	Plasmid construction (pMS383)
KPO-12169	AAATAACGAAATATTAAGGAAATAAAATGAAAAC	Plasmid construction (pMS384)
KPO-12170	TATTTCGTTATTTAAGGTGTGAAAAGG	Plasmid construction (pMS384)
KPO-12217	AGTTCTTTCTTCTCCTTCCGTGGATTTGGGCACCTTCGGGTGCCCCTTT TTAGATCCGGTGATTGATTGAGC	Plasmid construction (pMS390)
KPO-12218	CGGAAGGAGAAGAAAGAACT CCACACATTATACGAGCCGA	Plasmid construction (pMS390)
KPO-12219	AACCACCTATTCTATTTTCTCTTAGAGAACTTGAGCGCGGCTTCGGTCG CGCTCTTCTTTGATCCGGTGATTGATTGAGC	Plasmid construction (pMS391)
KPO-12220	AGAAAATAGAATAGGTGGTTCCACACATTATACGAGCCGA	Plasmid construction (pMS391)
pBAD-ATGrev	GGTTAATTCCTCCTGTTAGC	Plasmid construction

		(pEVS protein)
pZE-Stop-XbaI	TAATCTAGAGGCATCAAATAAAACGA	Plasmid construction (pEVS protein)
**Oligonucleotides for northern blot probing**		
KPO-00243	TTCGTTTCACTTCTGAGTTCGG	5S oligoprobe
KPO-02077	CCGCGAAAAGTAGGTTGTTTC	Vcr229 oligoprobe
KPO-02235	AAATCCATGGAAGGAGAAGGAAG	CisR oligoprobe
KPO-02683	GTGCTTAATCGTCAGCTTGTAAC	TarB oligoprobe
KPO-05415	CAACGGGAGAGAAAACGGTT	Vssrna24 oligoprobe
KPO-08374	ATTCTTCCTTCTCCTTCCATGG	CisR riboprobe
KPO-08375	GTTTTTTTAATACGACTCACTATAGGGAGGAAAAAAGGGGCACC CGAAGG	CisR riboprobe
**Oligonucleotides for qPCR**		
KPO-00496	GTTTGTACTTTACCGAACGC	P*vqmR* qPCR
KPO-02378	GGTAACCCAGAAACTACCACTG	*recA* qPCR
KPO-02379	CACCACTTCTTCGCCTTCTT	*recA* qPCR
KPO-07712	CGCCAATTATGTCGGTTTC	P*vqmR* qPCR
KPO-08732	GCATTTGCTAACCAAGCAC	*cep* qPCR
KPO-08733	AAGCGCAATCACCGTATC	*cep* qPCR
KPO-09026	TGTCTTATTAGTCATTTATTCGATTGAT	P*cisR* qPCR
KPO-09027	GGCTGGCGATAATTCATAGCAA	P*cisR* qPCR
KPO-09561	GCAATCTCTACAAGACCCATT	*hfq* qPCR
KPO-09562	CGATCTGACCTTGCAGTTT	*hfq* qPCR
**Oligonucleotides for qRT-PCR**		
KPO-00584	GCAAGGTCAGATCGAATCATTTG	*hfq* qRT-PCR
KPO-00585	GGTGGCTAACTGGACGAG	*hfq* qRT-PCR
KPO-02378	GGTAACCCAGAAACTACCACTG	*recA* qRT-PCR
KPO-02379	CACCACTTCTTCGCCTTCTT	*recA* qRT-PCR
KPO-02745	CATTCTTGGTGATCTCATGATAAGG	*toxT* qRT-PCR
KPO-02746	CATTTACCACTTCAGAAAGGACAG	*toxT* qRT-PCR
KPO-08374	ATTCTTCCTTCTCCTTCCATGG	*cisR* qRT-PCR

KPO-09656	AAAAAAGGGGCACCCGAAGG	*cisR* qRT-PCR
KPO-02747	AAGCAGTCAGGTGGTCTTATG	*ctxA* qRT-PCR
KPO-02748	ACAAATCCCGTCTGAGTTCC	*ctxA* qRT-PCR
KPO-08732	GCATTTGCTAACCAAGCAC	*cep* qRT-PCR
KPO-08733	AAGCGCAATCACCGTATC	*cep* qRT-PCR
**Oligonucleotides used for DNase I footprinting, EMSAs and *in vitro* transcription**		
P*vca0224* F	GGCTGCGAATTCGTTTAATTTCACTGATTATTTATTGTGCTC	P*vca0224* generation
P*vca0224* R	GCCCGAAGCTTGTATTTGTAACAATTTATGGCTGGCGAT	P*vca0224* generation
**Oligonucleotides used for rRNA depletion**		
16S_1	CCGCTCGACTTGCATGTGTTAAGCATGCCGACAGCGTTCG	rRNA depletion
16S_2	CCCATTGTGCAAGATTCCCTACTGCTGCCTCCCGT	rRNA depletion
16S_3	ACCGCGGCTGCTGGCACGGAGT	rRNA depletion
16S_4	ACGGCGTGGACTACCAGGGTAT	rRNA depletion
16S_5	TCCACATGCTCCACCGCTTGTGCGGGCCCCCG	rRNA depletion
16S_6	ACCCAACATCTCACAACACGAGCTGACGACA	rRNA depletion
16S_7	GGGCAGTGTGTACAAGGCCCGGGA	rRNA depletion
16S_8	AAGGAGGTGATCCAGCCGCAG	rRNA depletion
23S_1	ACCTTTCCCTCACGGTACTGGTTCGCTATCGGTCA	rRNA depletion
23S_2	AGTCGCTGGCTCATTATACAAAAGGTACGCCGTCACC	rRNA depletion
23S_3	TCGGGGAGAACCAGCTATCTCCGGGTTTGATTGGC	rRNA depletion
23S_4	GTGGCTGCTTCTAAGCCAACATCCTG	rRNA depletion
23S_5	GGGTACAGGAATATTAACCTGATTTCCATCGACTACGCC	rRNA depletion
23S_6	CACCTGTGTCGGTTTGGGGTACGGT	rRNA depletion
23S_7	TCGTGCGGGTCGGAACTTACCCGACAAG	rRNA depletion
23S_8	GAGCCGACATCGAGGTGCCAAACA	rRNA depletion
23S_9	CGGCGGATAGGGACCGAACTGTCTCACGAC	rRNA depletion
23S_GN1	CACGTCCTTCATCGCCTTTTACTGCCAAGGCATCC	rRNA depletion
23S_GN2	CCACACCCGGCCTATCAACGTGGTGGTCTTCGACG	rRNA depletion
KPO-09290	CTACTCTCACATGGGGAGACCCCACACTACC	rRNA depletion
KPO-09291	CGTTTCACTTCTGAGTTCGGGATGGAATCAGGTGGGTCC	rRNA depletion

Supplemental References

1. Hu D*, et al.* (2016) Origins of the current seventh cholera pandemic. *Proc Natl Acad Sci U S A* 113(48):E7730-E7739.
2. Corpet F (1988) Multiple sequence alignment with hierarchical clustering. *Nucleic acids research* 16(22):10881-10890.
3. Hoyos M, Huber M, Förstner KU, & Papenfort K (2020) Gene autoregulation by 3'UTR-derived bacterial small RNAs. *Elife* 9:e58836.
4. Siemers M, Lippegaus A, & Papenfort K (2024) ChimericFragments: computation, analysis and visualization of global RNA networks. *NAR Genom Bioinform* 6(2):lqae035.
5. Mann M, Wright PR, & Backofen R (2017) IntaRNA 2.0: enhanced and customizable prediction of RNA–RNA interactions. *Nucleic acids research* 45(W1):W435-W439.
6. Thelin KH & Taylor RK (1996) Toxin-coregulated pilus, but not mannose-sensitive hemagglutinin, is required for colonization by Vibrio cholerae O1 El Tor biotype and O139 strains. *Infect Immun* 64(7):2853-2856.
7. Svenningsen SL, Tu KC, & Bassler BL (2009) Gene dosage compensation calibrates four regulatory RNAs to control Vibrio cholerae quorum sensing. *The EMBO journal* 28(4):429-439.
8. Peschek N, Hoyos M, Herzog R, Forstner KU, & Papenfort K (2019) A conserved RNA seed-pairing domain directs small RNA-mediated stress resistance in enterobacteria. *EMBO J* 38(16):e101650.
9. Venkat K*, et al.* (2021) A dual-function RNA balances carbon uptake and central metabolism in Vibrio cholerae. *The EMBO journal* 40(24):e108542.
10. Huber M*, et al.* (2022) An RNA sponge controls quorum sensing dynamics and biofilm formation in Vibrio cholerae. *Nat Commun* 13(1):7585.
11. Corcoran CP*, et al.* (2012) Superfolder GFP reporters validate diverse new mRNA targets of the classic porin regulator, MicF RNA. *Molecular microbiology* 84(3):428-445.
12. Waters CM & Bassler BL (2006) The Vibrio harveyi quorum-sensing system uses shared regulatory components to discriminate between multiple autoinducers. *Genes Dev* 20(19):2754-2767.
13. Dunn AK, Millikan DS, Adin DM, Bose JL, & Stabb EV (2006) New rfp- and pES213-derived tools for analyzing symbiotic Vibrio fischeri reveal patterns of infection and lux expression in situ. *Appl Environ Microbiol* 72(1):802-810.
14. Skorupski K & Taylor RK (1996) Positive selection vectors for allelic exchange. *Gene* 169(1):47-52.
15. Kolb A, Kotlarz D, Kusano S, & Ishihama A (1995) Selectivity of the Escherichia coli RNA polymerase E sigma 38 for overlapping promoters and ability to support CRP activation. *Nucleic acids research*, *23*(5), 819–826.
